# An engineered Japanese encephalitis virus mRNA-lipid nanoparticle immunization induces protective immunity in mice

**DOI:** 10.3389/fmicb.2024.1472824

**Published:** 2024-11-11

**Authors:** Jiayang Zhu, Caiying He, Yusha Liu, Min Chen, Jiayi Zhang, Dong Chen, Hongxia Ni, Jinsheng Wen

**Affiliations:** ^1^School of Basic Medical Sciences, Health Science Center, Ningbo University, Ningbo, China; ^2^Wenzhou Central Blood Station, Wenzhou, China; ^3^Key Laboratory of Laboratory Medicine, Ministry of Education, Zhejiang Provincial Key Laboratory of Medical Genetics, College of Laboratory Medicine and Life Sciences, Wenzhou Medical University, Wenzhou, China; ^4^Ningbo Municipal Center for Disease Control and Prevention, Ningbo, China

**Keywords:** Japanese encephalitis virus, Zika virus, mRNA vaccine, recombinant adenovirus, antibody-dependent enhancement

## Abstract

**Introduction:**

Japanese encephalitis virus (JEV) and Zika virus (ZIKV) pose a severe threat to human health. Our previous research results, as well as those of other research groups, indicated that antibodies (Abs) induced by JEV infection or JEV vaccine vaccination could enhance ZIKV infection *in vitro* and exacerbate the mortality of ZIKV-infected mice, vice versa, which is known as antibody-dependent enhancement (ADE). Although studies on other flaviviruses revealed that altering the amino acid residues located in the fusion loop (FL) of envelope (E) protein can reduce the level of flavivirus-cross-reactive Abs, thereby abating the ADE of heterologous flavivirus infection, it is unclear whether this strategy is equally applicable to JEV.

**Methods:**

In this study, we constructed recombinant adenoviruses and nucleotide-modified mRNA-lipid nanoparticle (LNP) encoding JEV wild-type E protein or E protein mutant (designated as Ad5-JEV-E^WT^ and Ad5-JEV-E^mut^; JEV-E^WT^ mRNA-LNP, and JEV-E^mut^ mRNA-LNP). We evaluated the immunogenicity of these vaccine candidates in mice and the capacity of vaccine-immune mouse sera to neutralize JEV infection or mediate ADE of ZIKV infection *in vitro* and *in vivo*.

**Results:**

Ad5-JEV-E^mut^ or JEV-E^mut^ mRNA-LNP immunization induced ZIKV-cross-reactive Ab response which is dramatically lower than that induced by Ad5-JEV-E^WT^ and JEV-E^WT^ mRNA-LNP, respectively. The levels of JEV-neutralizing Abs induced by Ad5-JEV-E^mut^ or JEV-E^mut^ mRNA-LNP are comparable to that induced by Ad5-JEV-E^WT^ and JEV-E^WT^ mRNA-LNP, respectively. The ability of Abs induced by Ad5-JEV-E^mut^ to enhance ZIKV infection *in vitro* is attenuated as compared with that induced by Ad5-JEV-E^WT^. Moreover, JEV-E^mut^ mRNA-LNP immunization elicited potent T cell response similar to JEV-E^WT^ mRNA-LNP in mice. Mice immunized with each mRNA-LNP exhibited lower level of serum viral load than the mock-immunized mice post JEV challenge. Mice receiving JEV-E^WT^ mRNA-LNP-immune mouse sera exhibited ADE post ZIKV challenge whereas passively transferred JEV-E^mut^ mRNA-LNP-immune mouse sera did not lead to obvious ADE of ZIKV infection in recipient mice. Most importantly, maternally acquired Abs did not enhance the mortality of 1-day-old neonates born to JEV-E^mut^ mRNA-LNP-immunized mice post ZIKV challenge.

**Discussion:**

These results suggest that optimizing the FL sequence of JEV could significantly reduce the level of JEV/ZIKV-cross-reactive Abs and abrogate the ADE of ZIKV infection, providing a promising strategy to develop effective and safety JEV vaccine.

## 1 Introduction

Japanese encephalitis virus (JEV), as a mosquito-borne pathogen belonging to the genus flavivirus within the family *Flaviviridae*, is identified as a primary etiological agent of viral encephalitis (Sharma et al., 2021). As reported by the World Health Organization (WHO) in 2019, JEV accounts for approximately 68,000 annual cases globally (Campbell et al., 2011). JEV is mainly transmitted via *Culex tritaeniorhynchus* bite and predominantly endemic in 24 countries/territories including China, Japan, South Korea, etc. (Kumar et al., 2021). It was estimated that around three billion individuals are at the risk of JEV infection (Liu et al., 2020). Patients infected with JEV can show a variety of symptoms, from mild fever to severe hemorrhagic, encephalitic manifestations or even death (Huang et al., 2021). Previous studies indicated that the mortality rate for patients with Japanese encephalitis ranges from 20 to 30%. Furthermore, among the survivors, 30 to 50% will experience permanent neurological sequelae (Campbell et al., 2011; Solomon et al., 2002). To date, four types of JEV vaccines are issued for use: the mouse brain-derived inactivated JEV vaccine, the Vero cell-derived inactivated JEV vaccine, the cell culture-derived live-attenuated JEV vaccine, and the live recombinant (chimeric) vaccine (Hills et al., 2014; Batchelor and Petersen, 2015). Same to JEV, Zika virus (ZIKV) belongs to the genus flavivirus, family *Flaviviridae*. By July 2019, the cases of ZIKV infection were reported among 87 countries/territories (Fong and Chu, 2022). Different to JEV, the transmission vectors for ZIKV are *Aedes aegypti* and *Aedes albopictus* mosquitoes (Westaway et al., 1985). In addition to vector-borne transmission via mosquito bites, ZIKV can be transmitted sexually (Govero et al., 2016); moreover, vertical transmission of ZIKV can occur from mother to fetus during pregnancy, leading to congenital Zika syndromes, including fetal/newborn microcephaly (Rubin et al., 2016; Rasmussen et al., 2016; Mlakar et al., 2016). Roughly 80% of adults infected with ZIKV are asymptomatic, with the remaining patients exhibiting symptoms ranging from mild non-specific symptoms such as fever, joint and muscle pain, to severe neurological disorders such as Guillain–Barré syndrome (Musso et al., 2014; Cao-Lormeau et al., 2016; Dirlikov et al., 2016; Schuler-Faccini et al., 2016). Currently, there are no specific therapeutic agents or vaccines available for the treatment or prevention of ZIKV infection.

The phenomenon of antibody-dependent enhancement (ADE) refers to a scenario where, under certain conditions, one virus infection can be enhanced by antibodies (Abs) induced by a related virus (Bournazos et al., 2020). ADE in dengue virus (DENV) research field was first described by Halstead et al. (1973a,b). They observed that individuals with pre-existing immunity to one serotype of DENV are more likely to develop severe forms of the disease, such as dengue shock syndrome and dengue hemorrhagic fever, upon subsequent infection with a heterologous serotype (Watanabe et al., 2015; Muhammad Azami et al., 2020). Up to date, ADE was widely identified among many flaviviruses of the family *Flaviviridae* (Bardina et al., 2017; Chen et al., 2020; He et al., 2020; Zhang et al., 2020; Katzelnick et al., 2017; Katzelnick et al., 2020). For instance, mice receiving sera from patients infected with West Nile virus (WNV) exhibited a markedly higher mortality rate than mice given with naïve sera upon subsequent ZIKV infection (Bardina et al., 2017). In addition, transfer of sera obtained from DENV-infected patients significantly increased the mortality of ZIKV-infected mice (Bardina et al., 2017). Our recent study also indicated that 1-day-old pups born to JEV-immunized mice exhibited an increased mortality and elevated viremia than the newborns born to the naïve mothers after ZIKV attack (Chen et al., 2020). Moreover, our findings were subsequently confirmed by the experimental results of other labs (He et al., 2020; Zhang et al., 2020). Similarly, serum Abs from ZIKV-immunized mice could cross-recognize JEV antigen and increased the mortality of recipient mice post JEV challenge (Chen et al., 2020). The ADE phenomenon of DENV through animal experiments was confirmed by a clinical cohort study (Katzelnick et al., 2017). The results of another clinical cohort study by this research group also revealed that individuals infected with ZIKV are more likely to develop severe disease after infecting with DENV (Katzelnick et al., 2020). Considering that many flaviviruses (particularly JEV and ZIKV) have the overlapping epidemic areas and the fact that JEV vaccines is widely used among populations in multiple countries/territories, the ADE issue between JEV and ZIKV is of particular concern. Therefore, developing or optimizing flavivirus vaccine is a necessary measure.

The envelope (E) protein of flavivirus is located in the viral membrane and plays a pivotal role in the process of flavivirus infecting host cell. E protein could facilitate virus to bind to the receptor of susceptible cell and mediate the membrane fusion between virus and cell (Zhang et al., 2017). In fact, the extracellular part of E protein is composed of three domains: Domain I (EDI), Domain II (EDII), and Domain III (EDIII). The B-cell epitopes are predominantly located in EDI and EDIII (Tabata et al., 2023). EDII mainly stabilizes the homodimeric configuration of E protein and harbors a highly conserved fusion loop (FL) sequence among flaviviruses, mediating virus-cell membrane fusion (Chiou et al., 2012; Chiou et al., 2008). Previous studies reported that the FL sequence contains a critical epitope that is involved in the induction of flavivirus-cross-reactive Abs, resulting in the ADE of heterologous flavivirus infection (de Alwis et al., 2014; Gunawardana and Shaw, 2018; Vogt et al., 2011). Recently, a number of studies have demonstrated that replacing the different amino acid sites of FL could reduce the production of flavivirus-cross-reactive Abs, and even alleviate ADE between flaviviruses (Vogt et al., 2011; Crill et al., 2007; Berneck et al., 2020; Zhang et al., 2006; Rockstroh et al., 2015). However, it is unclear whether optimizing the FL sequence of JEV can reduce the production of Abs that have ADE effects on ZIKV infection.

In the present study, to clarify whether optimizing the FL sequence of JEV E protein can reduce the production of ZIKV-cross-reactive Abs and attenuate the ADE activity of JEV-induced Abs against ZIKV infection, we generated recombinant adenoviruses and mRNA-lipid nanoparticle (mRNA-LNP) on the basis of the amino acid sequences of JEV wild-type E protein and E protein mutant. Our data show here, (1) the ability of Abs induced by Ad5-JEV-E^mut^ or JEV-E^mut^ mRNA-LNP to cross-recognize ZIKV antigen is significantly reduced; (2) the neutralizing activity of Ad5-JEV-E^mut^-immune or JEV-E^mut^ mRNA-LNP-immune mouse sera against JEV infection is comparable to the Abs induced by Ad5-JEV-E^WT^ and JEV-E^WT^ mRNA-LNP, respectively; (3) the ability of Abs induced by Ad5-JEV-E^mut^ to mediate ADE of ZIKV infection *in vitro* is attenuated; (4) JEV-E^mut^ mRNA-LNP immunization induced a comparable level of CD8^+^ T cell response to JEV-E^WT^ mRNA-LNP; (5) JEV-E^mut^ mRNA-LNP immunization can protect mice against JEV challenge, characterized by the significant reduction of serum viral load; (6) passive transfer of the JEV-E^mut^ mRNA-LNP-immune mouse sera does not increase the mortality of recipient mice post ZIKV challenge; (7) maternally acquired anti-JEV Abs does not increase the mortality of pups born to JEV-E^mut^ mRNA-LNP-immunized mice after ZIKV challenge.

## 2 Materials and methods

### 2.1 Cell lines, viruses and reagents

Vero cell was purchased from American Type Culture Center (ATCC) and maintained in RPMI-1640 medium supplemented with 10% fetal bovine serum and 1% penicillin/streptomycin. HEK293T cell was obtained from ATCC and cultured using MEM medium supplemented with 10% fetal bovine serum and 1% penicillin/streptomycin. Both JEV (strain ZJ14-52, GenBank Accession No. MK558811) and ZIKV (strain Zhejiang04, GenBank Accession No. KX117076.1) were propagated in Vero cells and the viral stocks were tittered using plaque-forming assay (PFA) as described below. The viral titer was expressed as plaque-forming unit (PFU)/mL. HRP-conjugated goat anti-mouse IgG and FITC-conjugated goat anti-mouse IgG were purchased from Proteintech (Chicago, America). 3,3′,5,5′-Tetramethylbenzidine (TMB) was purchased from Macklin (Shanghai, China). Methyl cellulose M450 was purchased from Ourchem (Beijing, China). Isopropyl-β-D-thiogalactoside (IPTG) was purchased from Solarbio (Beijing, China). Polyetherimide (PEI) transfection reagent was purchased from GlpBio (California, United States).

### 2.2 Mice

Six-week-old male and female wild-type (WT) C57BL/6 mice were purchased from the Animal Model Research Center at Nanjing University, China, and were maintained and bred in the Animal Center of Ningbo University, China. The experiments were performed in strict accordance with the animal welfare act and public health service policy on humane care and use of laboratory animals and were approved by the ethical committee of Ningbo University (No. 2020-55). Sample sizes were estimated on the basis of our previous studies. Animal experiments were randomized but not blinded.

### 2.3 Peptide synthesis

Three previously identified H-2^b^-restricted CD8^+^ T cell epitopes in ZIKV (ZIKV-E_4–12_, ZIKV-E_57–65_, and ZIKV-E_420–428_) and their variants in JEV (JEV-E_4–12_, JEV-E_57–65_, and JEV-E_416–424_) (Chen et al., 2020) were synthesized by ChinaPeptides with a purity >95% (Supplementary Table S1).

### 2.4 Expression of viral proteins

The genes (optimized for *E. coli* codon usage) encoding the extracellular domain of E protein of JEV (E_406_, aa 1-406) and ZIKV (E_410_, aa 1-410) were synthesized by GeneWiz (Suzhou, China) and cloned into the pET21a expression vectors, each featuring a 6 × His tag at the 3′ terminal of gene sequence. pET21a-JEV-E_406_ and pET21a-ZIKV-E_410_ were, respectively transformed into *E. coli* BL21 strain, and protein expression was induced using IPTG at a final concentration of 1 mM. The recombinant JEV-E_406_ and ZIKV-E_410_ proteins were purified using a Ni-NTA (TransGen Biotech, Beijing, China), followed by further concentrated through a 10 kDa ultrafiltration tube (Millipore, Darmstadt, Germany). The protein purity was assessed by SDS-PAGE and the protein concentration was quantified using a BCA kit (TransGen Biotech).

### 2.5 Construction of recombinant adenoviruses

The genes (optimized for *Homo sapiens* codon usage) encoding the complete amino acid sequences of wild-type E protein (E^WT^, 500 aa) or E protein mutant (E^mut^, 500 aa, with W101A and F108A amino acid substitutions in the FL sequence) of JEV were synthesized by GeneWiz and cloned into the pCAGGS plasmid, resulting in recombinant plasmids pCAGGS-JEV-E^WT^ and pCAGGS-JEV-E^mut^. After dual enzyme digestion of the recombinant plasmid and pShuttle-CMV plasmid, the purified gene fragment was ligated with the empty pShuttle-CMV plasmid and transformed into competent bacteria Stbl3 strain to produce recombinant pShuttle-CMV plasmids (pShuttle-CMV-JEV-E^WT^ or pShuttle-CMV-JEV-E^mut^). The linearized plasmid pShuttle-CMV-JEV-E^WT^ or pShuttle-CMV-JEV-E^mut^ was electroporated into competent *E. coli* BJ5183 strain containing a human adenovirus type 5 (Ad5) genomic backbone plasmid (pAdEasy-1) for homologous recombination, and the recombinant Ad5 plasmids (Ad5-JEV-E^WT^ and Ad5-JEV-E^mut^) were screened out. The recombinant Ad5 plasmids were transfected into HEK293T cell to produce recombinant Ad5 which was then tittered by checking the GFP-positive cell using confocal microscope on the basis of Vero cell. The titer of recombinant Ad5 was expressed as infectious unit (IFU) per ml. Recombinant adenoviruses were used to infect Vero cells and the expression of JEV-E protein in the cell supernatant was evaluated using WB. JEV-E_406_-immune mouse sera were used as the primary antibody and HRP-conjugated goat anti-mouse IgG mAb worked as the secondary antibody.

### 2.6 Generation of mRNA sequences encoding JEV-E protein *in vitro*

To prepare a mRNA sequence encoding JEV-E protein *in vitro*, we designed a DNA sequence consisting of T7 promoter, a 5′ UTR sequence, a Kozak sequence, a signal peptide sequence, gene sequence encoding JEV-E^WT^ or JEV-E^mut^ (W101A and F108A substitutions in the FL sequence), a 3′ UTR sequence, and a poly A tail from 5′ terminal to 3′ terminal, respectively (Supplementary Table S2). The DNA sequence was engineered into an optimized sequence with as much as more codons containing A or G (Supplementary Table S2) and cloned into the eukaryotic plasmid pBluescript II KS (+) to produce recombinant plasmids pBluescript II KS (+)-JEV-E^WT^ and pBluescript II KS (+)-JEV-E^mut^. Using the linearized pBluescript II KS (+)-JEV-E^WT^ or pBluescript II KS (+)-JEV-E^mut^ as template, RNA was produced using an *in vitro* transcription kit after supplementing with N1-methyl-pseudouridine and other components. Finally, the 5′ terminal of RNA was capped using capping reagent kit (Novoprotein, Suzhou, China) to generate functional JEV-E^WT^ mRNA and JEV-E^mut^ mRNA.

### 2.7 Transfection of 293T cells with mRNA

293T cells were seeded to cell culture plate (10 cm^2^) and incubated for 24 h in a 37°C/5%CO_2_ incubator. Next day, the cell supernatant was discarded and MEM medium without FBS was added to the cells. Four hours later, 10 μg mRNA was diluted in PBS to reach 0.5 mL. 0.5 mL PEI (20 μg) was added to diluted mRNA and incubated for 20 min at room temperature. The PEI/mRNA mixture was added to cells and transfected for 4 h. After discarding the transfection reagent, complete MEM medium (with 10% FBS) was added to the cells and incubated for 2 days in the incubator. The cell supernatant was harvested for detecting JEV-E protein using WB. JEV-E_406_-immune mouse sera were used as the primary antibody and HRP-conjugated goat anti-mouse IgG mAb worked as the secondary antibody.

### 2.8 Production of mRNA-lipid-nanoparticle

The purified mRNA was diluted into citrate buffer (pH4, 50 mM) to form the aqueous phase with a final concentration of 170 μg/mL. Briefly, 4-(N,N-dimethylamino) butyric acid (dilinoleic) methyl ester (Dlin-MC3-DMA; cationic lipid), Diethyl phosphatidylcholine (DSPC; non-cationic lipid), Cholesterol (sterol), and 1, 2-dimyristoyl-rac-glycero-3-methoxypolyethylene glycol-2000 (DMG-PEG2000; PEG-modified lipid) were dissolved into ethanol to make the organic phase, reaching the final concentration of 6.25 mM, 1.25 mM, 4.8125 mM, 0.1875 mM, respectively. The aqueous phase and the organic phase were mixed at a volume ratio of 3:1 and passed through a Microflow S microfluidic nanomedicine fabrication instrument at a flow rate of 12 mL/min to produce JEV-E^WT^ mRNA-LNP or JEV-E^mut^ mRNA-LNP. The zeta potential and polydispersity of mRNA-LNP were determined using particle size analyzer. The particle size of mRNA-LNP was measured using transmission electron microscope. The mRNA-LNP was cleaved using 2% Triton X-100 and the amount of released mRNA was measured for calculating the mRNA encapsulation rate.

### 2.9 Animal immunization

Recombinant adenovirus and JEV-E mRNA-LNP were used to immunize C57BL/6 mice. As for recombinant adenovirus immunization, 6-week-old male mice were subcutaneously (s.c.) injected with Ad5-JEV-E^WT^ or Ad5-JEV-E^mut^ (1 × 10^8^ IFU/mouse). The mice receiving PBS were used as control. Two weeks later, the mice were boosted with the same recombinant adenovirus. Two weeks after the second immunization, the mice were sacrificed and the mouse sera were collected for ELISA, plaque-reduction neutralization test (PRNT), and *in vitro* ADE assay. The mRNA-LNP was used to immunize adult (6-week-old) or young (1-week-old) mice. In one protocol, 6-week-old male or female mice were injected s.c. with JEV-E^WT^ mRNA-LNP or JEV-E^mut^ mRNA-LNP (5 or 50 μg mRNA/mouse) twice at a two-week interval. The mice receiving PBS were used as control. Two weeks after the second immunization, the male mice were sacrificed and the mouse sera were used for ELISA, PRNT, and passive transfer experiments. The splenocytes were used for ELISpot assay. The immunized female mice were mated with 10-week-old naïve male mice. Approximate 3 weeks later, the 1-day-old neonates born to the mRNA-LNP-immunized mice were injected s.c. with 1,000 PFU of ZIKV, and the mouse weight and mortality were recorded daily for 28 days. In another protocol, 1-week-old neonates were injected s.c. with mRNA-LNP (0.1 or 1 μg mRNA/mouse) and boosted with the same mRNA-LNP 2 weeks later. Two weeks after the second immunization, some immunized mice were sacrificed and the mouse sera were used for ELISA and PRNT; the remaining mice were injected s.c. with 1 × 10^5^ PFU of JEV. Three days later, the mice were sacrificed and the mouse sera were harvested for viral load detection using PFA.

### 2.10 ELISA

The antigen-reactive IgG in mouse sera (recombinant adenovirus-infected mice; mRNA-LNP-immunized adult mice; mRNA-LNP-immunized young mice) was detected using indirect ELISA. In brief, the high-affinity ELISA plates were coated with recombinant JEV-E_406_ or ZIKV-E_410_ proteins (20 μg/mL, 100 μL/well) and incubated for 1.5 h at 37°C. The plates were then blocked for 1.5 h at 37°C using 5% non-fat milk/PBS solution. Serially diluted mouse sera were added to the plates and incubated for 1.5 h at 37°C. HRP-conjugated Goat anti-mouse IgG (1:2,000 dilution) was added to the plate and incubated for 1.5 h at 37°C. The plate was developed with fresh TMB solution for 10 min at room temperature. The OD_450_ value of each well was measured using a microplate reader.

### 2.11 Plaque reduction neutralization test

The ability of recombinant adenovirus-immune or mRNA-LNP-immune mouse sera to neutralize JEV *in vitro* was evaluated on Vero cells using PRNT. In brief, Vero cells were seeded in each well (1 × 10^5^ cells/well) of 24-well cell culture plate and incubated for 24 h in a 37°C/5% CO_2_ incubator. Mouse sera (recombinant adenovirus-infected mice; mRNA-LNP-immunized adult mice; mRNA-LNP-immunized young mice) were 3-fold serially diluted in the well (240 μL/well) of 96-well culture plate, followed by addition of 50 μL of JEV solution (containing 50 PFU), and incubated for 1 h at 37°C. After discarding the supernatant of each well, the mixture of serum/virus was added to Vero cell and infected for 1 h at 37°C. After discarding the viral solution, the cells were overlaid with 1% methyl cellulose/medium and incubated for 3 days in a 37°C/5% CO_2_ incubator. The cells were fixed with 4% paraformaldehyde for 1 h at room temperature and stained with 0.5% crystal violet solution for 10 min at room temperature. The plaques of each well were counted and the NT_50_ (the highest serum dilution that can reduce viral plaques by 50%) titers were calculated.

### 2.12 ADE assay

The potential of mouse sera to enhance the infection of ZIKV was assessed on the basis of K562 cells. K562 cells were plated to each well (2 × 10^4^ cells/well) of U-bottom 96-well cell culture plates and incubated for 24 h in the 37°C/5% CO_2_ incubator. Mouse sera (Ad5-JEV-E^WT^-immune, Ad5-JEV-E^mut^-immune) were serially diluted in the well (50 μL/well) of 96-well plate and 50 μL of ZIKV (containing 1,000 PFU) were added to each well, and incubated at 37°C for 1 h. After centrifuged for 10 min at 2,000 rpm, the supernatant of K562 cells was discarded, followed by addition of the serum/virus mixture. After 1-h infection at 37°C, the viral solution was discarded and fresh medium was added to cells. After 24-h incubation, the cells were collected and fixed with 4% paraformaldehyde for 40 min at room temperature. The cells were then permeabilized with 0.5% Triton X-100/PBS for 40 min at temperature. The cells were incubated with viral antigen-specific antibody (ZIKV-E_410_-immune mouse serum, 1:200 dilution) for 1 h at room temperature, followed by incubation with FITC-labeled goat anti-mouse IgG mAb (1:1000 dilution) for 1 h at room temperature. The percentage of FITC-positive cells among each sample was determined using flow cytometry.

### 2.13 Mouse IFNγ ELISpot assay

Mouse splenocytes were plated to each well (1 × 10^6^/100 μL/well) of 96-well precoated ELISpot plate (DAKEWE, coated with anti-mouse IFNγ mAb), followed by addition of individual peptide (2 μg/well). Wells with cells but without peptide were served as negative control. After 24 h incubation in a 37°C/CO_2_ incubator, the plates were incubated sequentially with biotinylated anti-mouse IFN-γ mAb, HRP-conjugated streptavidin, and developed with 3-amino-9-ethylcarbazole color development solution. The colored spots of each well were counted and the results were expressed as the number of spot-forming cell (SFC) per 1 × 10^6^ mouse splenocytes. The presence of >20 SFCs/well with a stimulation index of >2 (index = ratio of test SFCs to control SFCs) was considered a positive response.

### 2.14 Passive transfer experiments

The effect of JEV-E mRNA-LNP-immune mouse sera on JEV or ZIKV infection *in vivo* was determined using passive transfer experiments. Different volume (3 or 15 μL) of either JEV-E^WT^ mRNA-LNP-immune mouse sera or JEV-E^mut^ mRNA-LNP-immune mouse sera were injected s.c. into 1-day-old mice. Two hours later, the mice were injected s.c. with 10 PFU of JEV or 1,000 PFU of ZIKV. The mouse weights and mortality were recorded daily for 14 days and 28 days, respectively.

### 2.15 Tissue viral loads detection

The titers of viral stock and the viral load in mouse sera were determined using PFA. Briefly, Vero cells are seeded to each well (1 × 10^5^ cells/well) of 24-well cell culture plate and incubated for 24 h in a 37°C/5% CO_2_ incubator. After discarding the cell supernatant, 240 μL of serially diluted viral stock or mouse serum was added to cells and the plate was incubated for 1 h at 37°C. After discarding the samples, the cells were overlaid with 1% Methyl cellulose/medium and incubated for 3–5 days in a 37°C/5% CO_2_ incubator. The cells were fixed with 4% paraformaldehyde for 1 h at room temperature and stained with 0.5% crystal violet solution for 10 min at room temperature. The numbers of plaque in each well were counted and the viral load in mouse serum was expressed as PFU/mL.

### 2.16 Statistical analysis

All data were analyzed using Prism 8 software (GraphPad Software, La Jolla, CA, United States) and expressed as the means ± SEM. The comparison between group means and survival data were analyzed using the Mann–Whitney test and a log-rank test, respectively. *p* < 0.05 was considered statistically significant.

## 3 Results

### 3.1 Production of recombinant adenovirus encoding JEV-E protein

**Figure 1:**
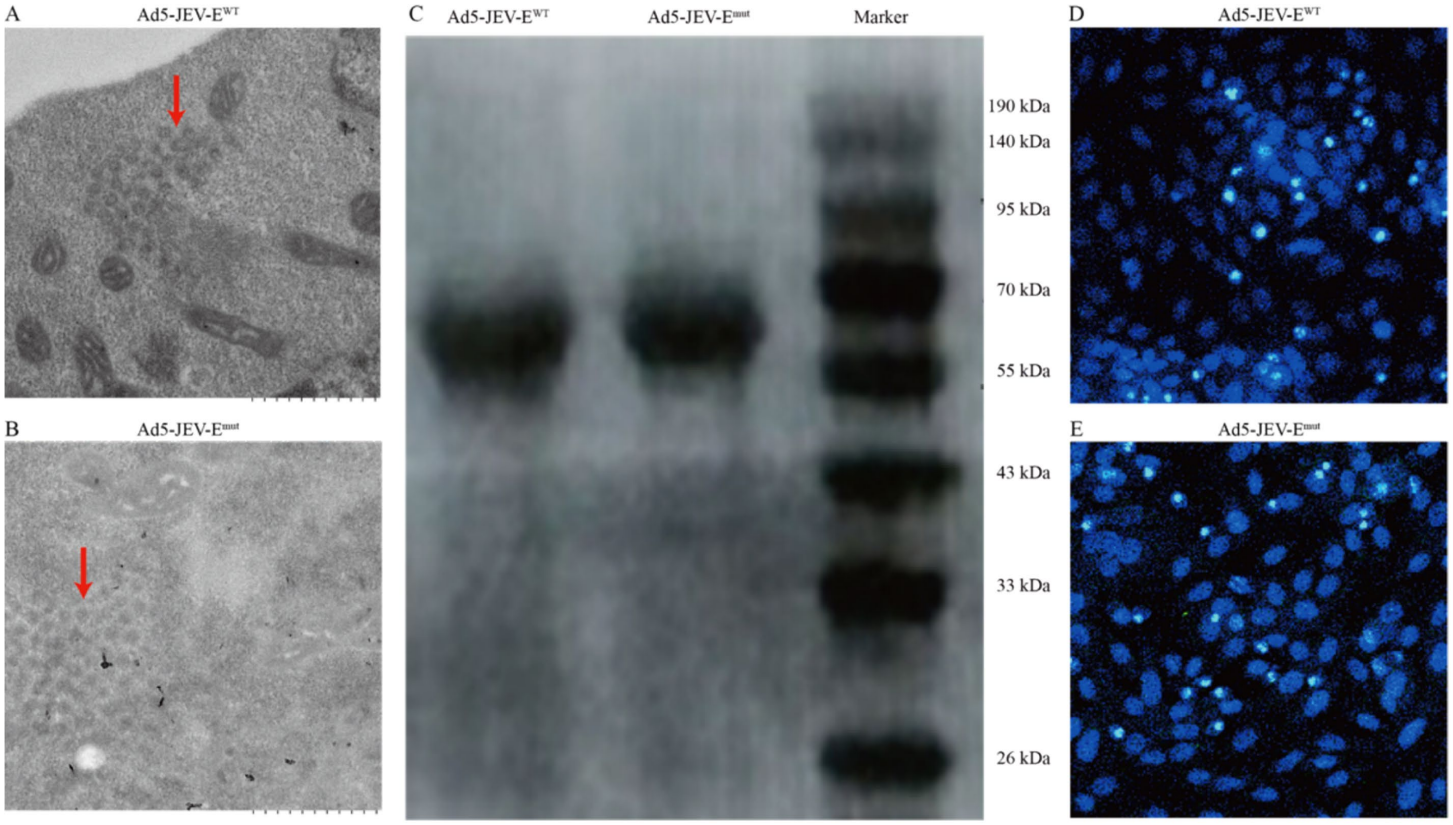
Generation of recombinant adenovirus encoding JEV-E protein. (A,B) 293T cells were transfected with recombinant adenovirus plasmids JEV-E^WT^ or JEV-E^mut^ and the viral particles of recombinant adenovirus in the cells were observed using transmission electron microscopy. (C) Vero cells were infected with recombinant adenoviruses Ad5-JEV-E^WT^ or Ad5-JEV-E^mut^ and the expression of JEV-E protein was determined using WB. (D,E) Vero cells were infected with recombinant adenoviruses Ad5-JEV-E^WT^ or Ad5-JEV-E^mut^ and the GFP-positive cells were observed using confocal microscopy.

To explore whether optimizing the FL sequence of the JEV-E protein affects the production of ZIKV-cross-reactive Abs, we initially designed the amino acid sequence of JEV-E protein mutant (JEV-E^mut^) by incorporating two amino acid substitutions to the wild-type E protein (JEV-E^WT^). With the assistance of shuttle plasmid, the genes encoding the JEV-E^WT^ or JEV-E^mut^ were successfully transferred into the human Ad5 genomic backbone plasmid to produce a recombinant adenovirus plasmid carrying the JEV-E^WT^ gene or JEV-E^mut^ gene. After transfecting the linearized recombinant adenovirus plasmid into 293T cells, recombinant adenovirus particles (having an average diameter of around 100 nm) were identified in the cytoplasm of cells using transmission electron microscopy (Figures 1A,B). We collected the cell supernatants and purified the recombinant adenoviruses Ad5-JEV-E^WT^ and Ad5-JEV-E^mut^. Two recombinant adenoviruses were sent to company GeneWiz for sequencing, and the inserted nucleotide sequences showed no nucleotide loss, insertion, or mutation as compared to our original sequences (data not shown). Both Ad5-JEV-E^WT^ and Ad5-JEV-E^mut^ were used to infect Vero cells and the presence of JEV-E protein in the cell supernatant was determined using WB (Figure 1C). Since the recombinant adenovirus carries the gene encoding GFP, the expression of GFP in the Vero cells infected with Ad5-JEV-E^WT^ or Ad5-JEV-E^mut^ was observed using confocal microscopy and the titer of recombinant adenovirus was determined by counting GFP-positive cells (Figures 1D,E).

### 3.2 Generation of JEV-E mRNA-LNP *in vitro*

**Figure 2:**
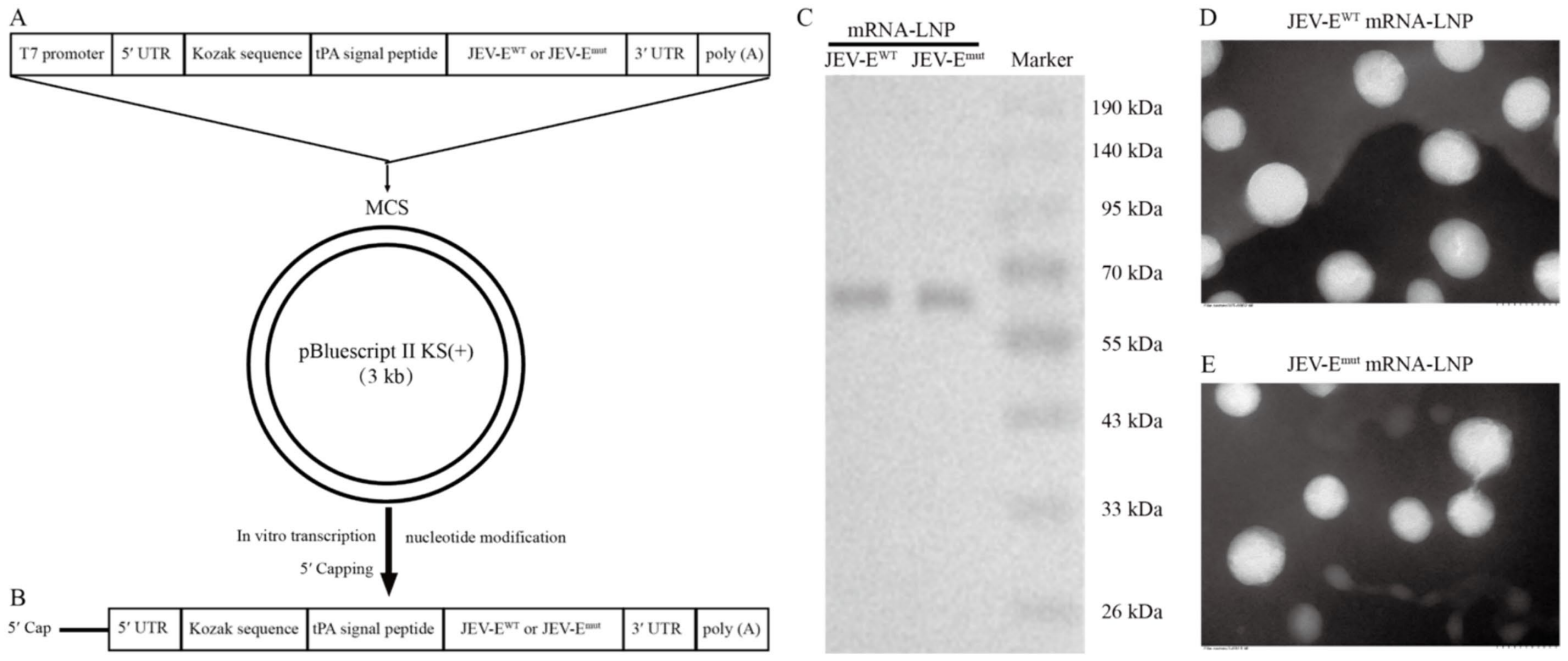
Generation of JEV-E mRNA-LNP. (A) The gene map of DNA used for *in vitro* transcription of RNA. (B) Transcription of RNA *in vitro* using linearized recombinant plasmid as template. (C) JEV-E^WT^ mRNA or JEV-E^mut^ mRNA was transfected into 293T cells using PEI transfection reagent and the expression of E protein was detected using WB. (D,E) The mRNA-LNP particle size was analyzed using transmission electron microscopy.

We further designed DNA sequence for producing mRNA *in vitro* and cloned it into pBluscript II KS (+) plasmid, resulting into pBluscript II KS (+)-JEV-E^WT^ and pBluscript II KS (+)-JEV-E^mut^ (Figure 2A). On the basis of linearized recombinant plasmid, we proceeded to generate mRNA encoding JEV-E^WT^ or JEV-E^mut^ with a length of 1875 bp (Figure 2B; Supplementary Figure S1). Following transfection of Vero cells with either JEV-E^WT^ mRNA or JEV-E^mut^ mRNA, the expression levels of the E protein were assessed using WB, displaying a similar expression proficiency (Figure 2C). We next encapsulated the mRNA within four components (cationic lipid, non-cationic lipid, cholesterol, and PEG-modified lipid) and produced the mRNA-LNP. The transmission electron micrograph showed that the mRNA-LNPs have an average diameter of around 100 nm (Figures 2D,E).

### 3.3 Optimizing the fusion loop sequence of JEV attenuates the production of ZIKV-cross-reactive antibodies

**Figure 3:**
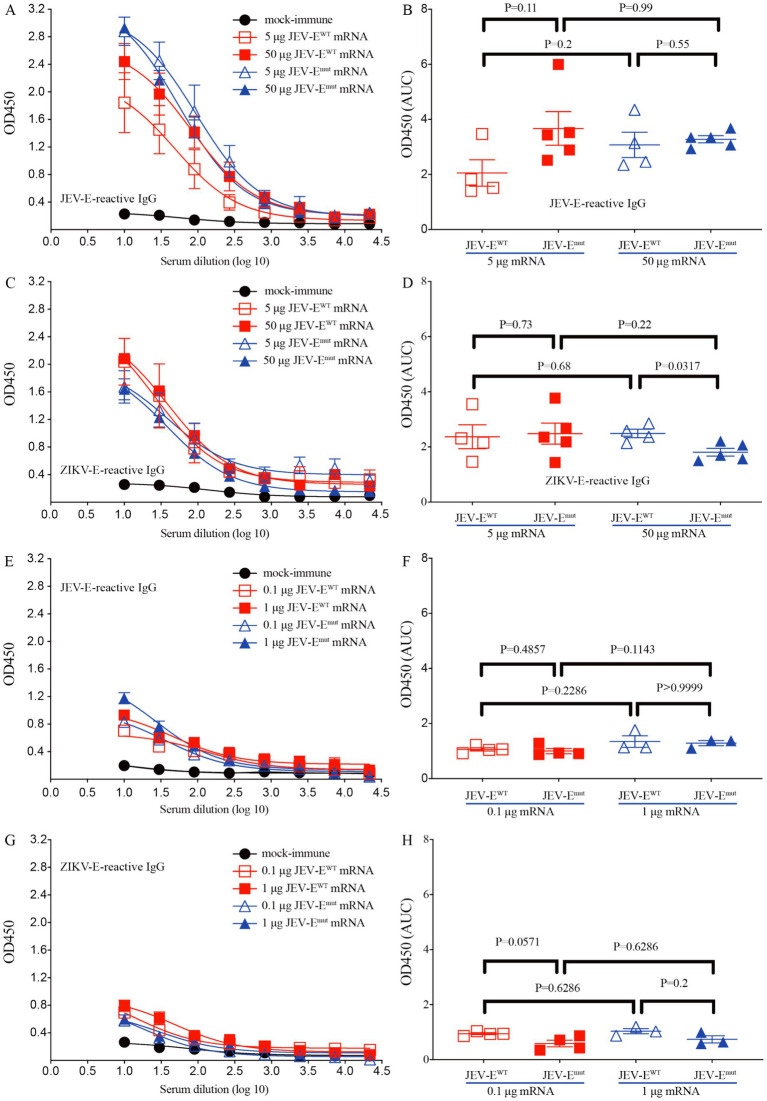
JEV-E^mut^ mRNA-LNP immunization induces low level of ZIKV-E-cross-reactive IgG response. Different doses of JEV-E^WT^ mRNA-LNP or JEV-E^mut^ mRNA-LNP (5 or 50 μg mRNA/adult mouse; 0.1 or 1 μg mRNA/young mice) were used to immunize adult (6-week-old) mice or young (1-week-old) mice twice as described in materials and methods. (A–D) Mouse sera were collected from mock-immune, JEV-E^WT^ mRNA-LNP-immune, and JEV-E^mut^ mRNA-LNP-immune adult mice. (E–H) Mouse sera were collected from mock-immune, JEV-E^WT^ mRNA-LNP-immune, and JEV-E^mut^ mRNA-LNP-immune young mice. The levels of JEV-E_406_- and ZIKV-E_410_-reactive IgG were measured using ELISA. Data are presented as the mean ± SEM. ^*^p < 0.05 by two-tailed Mann–Whitney *U* test. “AUC”: area under curve.

**Figure 4:**
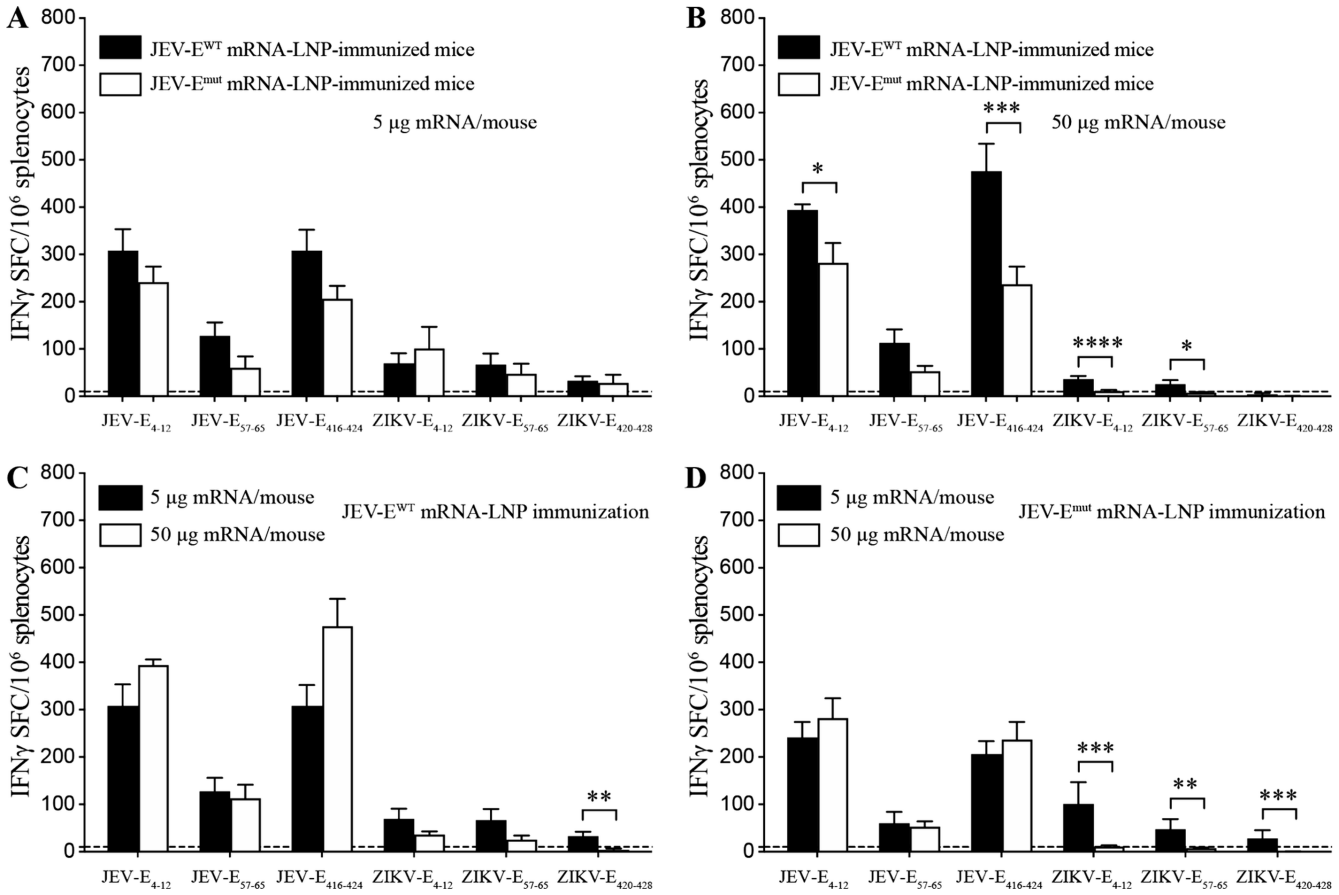
JEV-E mRNA-LNP immunization induces T cell response. **(A–D)** C57BL/6 mice were immunized with JEV-E^WT^ mRNA-LNP or JEV-E^mut^ mRNA-LNP as described in Materials and Methods. Twenty-eight days later, the splenocytes were stimulated *in vitro* with the indicated CD8^+^ T cell epitopes from JEV or the corresponding ZIKV variants. The frequencies of IFNγ-producing CD8^+^ T cells (SFC) were detected using IFNγ ELIspot assays. Data are presented as the mean ± SEM. ^*^p < 0.05, ^**^p < 0.01, and ^***^p < 0.001 by two-tailed Mann–Whitney *U* test.

We next immunized 6-week-old mice with recombinant adenoviruses Ad5-JEV-E^WT^ or Ad5-JEV-E^mut^, and found that each recombinant adenovirus immunization induced the production of JEV-E-reactive IgG (Supplementary Figures S2A,B), which could cross-recognize ZIKV-E protein. Interestingly, the ability of Ad5-JEV-E^mut^-immune mouse serum Abs to recognize ZIKV-E protein was significantly weaker than that of Ad5-JEV-E^WT^-immune mouse serum Abs (Supplementary Figures S2C,D). Next, 6-week-old mice were immunized with different doses of JEV-E^WT^ mRNA or JEV-E^mut^ mRNA in the form of LNP twice at a two-week internal, and the levels of JEV-E protein-reactive IgG and cross-reactive Abs against ZIKV-E protein were quantified using an ELISA. The results demonstrated that whether it is 5 μg or 50 μg, JEV-E^WT^ mRNA-LNP and JEV-E^mut^ mRNA-LNP induced a similar level of JEV-E-reactive antibody response (Figures 3A,B). However, 50 μg of JEV-E^mut^ mRNA induced a significantly lower level of ZIKV-E-cross-reactive antibody response compared to JEV-E^WT^ mRNA (Figures 3C,D). Considering 10-week-old mice resist JEV infection and have no obvious viremia post JEV infection whereas 5-week-old mice are susceptible to JEV infection and can maintain viremia for several days post JEV infection (data not shown), we then immunized 1-week-old young mice with different doses of JEV-E^WT^ mRNA-LNP or JEV-E^mut^ mRNA-LNP twice at a two-week interval and determined the JEV-E protein-reactive IgG in the mouse sera at 2 weeks post the second immunization. Similar Ab response against JEV-E protein and ZIKV-E protein were observed in mouse sera (Figures 3E–H). These data indicate that the substitution of amino acid residues among the FL of JEV can significantly attenuate the induction of ZIKV-E-cross-reactive Abs, which is not affected by mouse age. We next evaluate the cellular immune response induced by JEV-E mRNA-LNP immunization by detecting the frequencies of peptide-specific T cells in the splenocytes of JEV-E mRNA-LNP-immunized mice. The results show that 5 μg JEV-E^WT^ mRNA induced a similar T cell immune response with 5 μg JEV-E^mut^ mRNA while 50 μg JEV-E^WT^ mRNA induced a stronger T cell immune response than 50 μg JEV-E^mut^ mRNA (Figures 4A,B). But, 50 μg mRNA did not significantly increase T cell response than 5 μg mRNA (Figures 4C,D). These results indicate that substitution of amino acid residues in the FL sequence of JEV can significantly reduce the level of ZIKV-cross-reactive antibody response, but does not affect the humoral and cellular immune responses against JEV antigens.

### 3.4 Ad5-JEV-Emut-immune mouse serum abs mediate weak ADE of ZIKV infection *in vitro*

**Figure 5:**
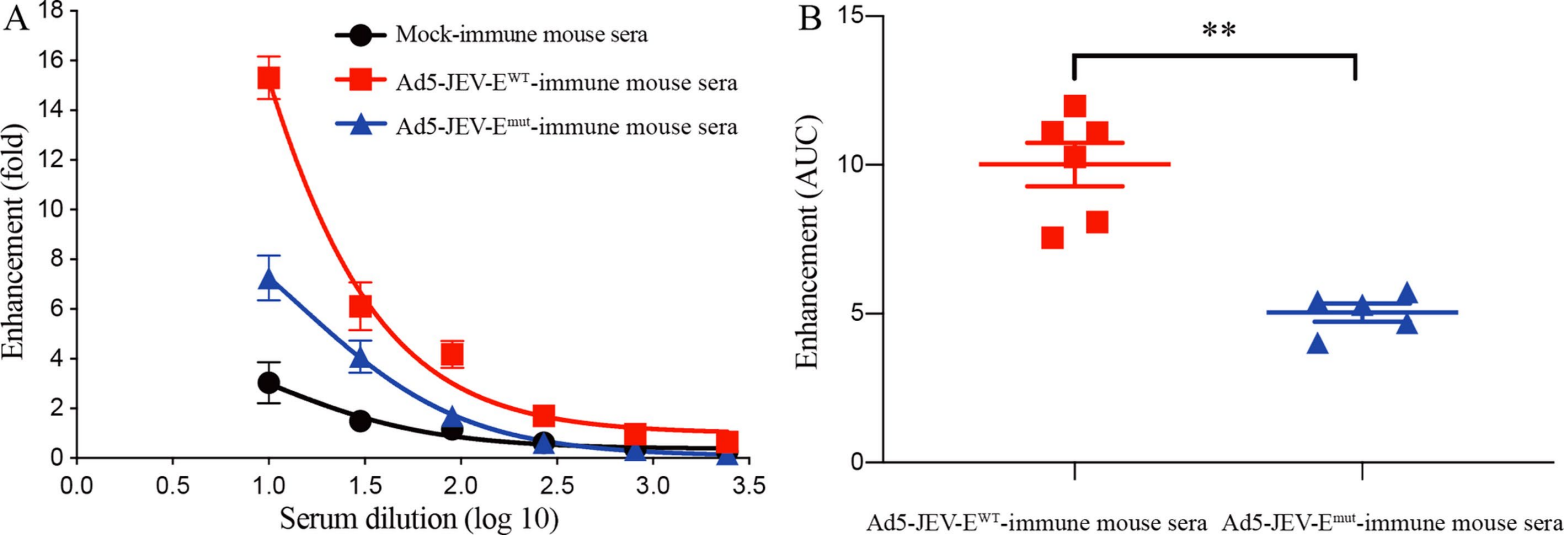
Antibodies induced by Ad5-JEV-E^mut^ mediate attenuated ADE of ZIKV infection *in vitro*. **(A,B)** K562 cells were infected with ZIKV at the absence or presence of diluted Ad5-JEV-E^WT^-immune mouse serum or Ad5-JEV-E^mut^-immune mouse serum. The percent infection of K562 cells were determined by detecting ZIKV antigen-positive cells using flow cytometry. Data are presented as the mean ± SEM. ^**^p < 0.01 by two-tailed Mann–Whitney *U* test.

Due to the resistance of K562 cells to ZIKV infection and its inability to be directly infected, the ZIKV-binding Abs can mediate the virus entry to cells by binding to the Fc receptors on the surface of K562 cells, making it a commonly used cell line for performing *in vitro* ADE assay (Chen et al., 2020). Therefore, we compared the differences in enhancing ZIKV infection *in vitro* between Ad5-JEV-E^WT^-immune mouse sera and Ad5-JEV-E^mut^-immune mouse sera based on K562 mice. As shown in Figures 5A,B, Ad5-JEV-E^WT^-immune mouse serum has a strong enhancing effect on ZIKV infection, and enhanced infection fold decreases continuously with serum diluted. When the serum is diluted at 1:10, the infection of ZIKV is increased by about 16 folds. Although Ad5-JEV-E^mut^-immune mouse serum still has an enhancing effect on ZIKV infection, there is a significant reduction compared to Ad5-JEV-E^WT^-immune mouse serum in total. When the serum is diluted at 1:10, it enhances ZIKV infection by about 8 folds.

### 3.5 Optimizing the fusion loop sequence of JEV does not affect the level of JEV-neutralizing antibodies

**Figure 6:**
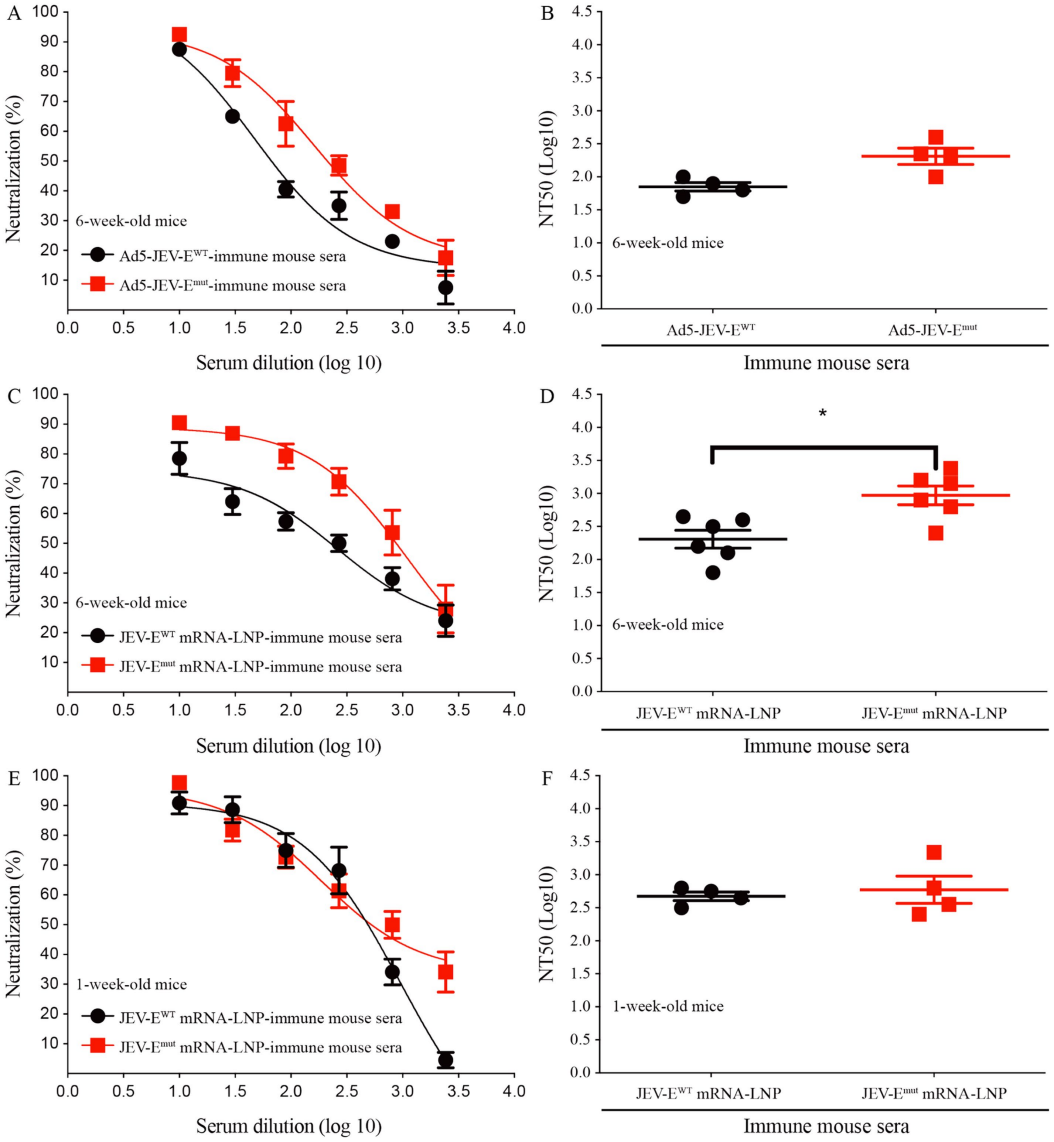
Optimizing the fusion loop sequence of JEV does not reduce the production of JEV-neutralizing antibodies. **(A–F)** Ad5-JEV-E^WT^-immune, Ad5-JEV-E^mut^-immune, JEV-E^WT^ mRNA-LNP-immune, JEV-E^mut^ mRNA-LNP-immune mouse sera were tested for neutralization of JEV infection of Vero cells using plaque-reduction neutralization test. Data are presented as the mean ± SEM. ^*^p < 0.05 by two-tailed Mann–Whitney *U* test.

In light of the aforementioned substitution of two amino acids within the FL region of the E protein were shown to attenuate the induction of ZIKV-cross-reactive Abs, we were then propelled to investigate the potential impact of such amino acid substitutions on the production of neutralizing Abs against JEV using PRNT. The results demonstrate that sera from mice immunized with Ad5-JEV-E^mut^ effectively neutralized JEV *in vitro*, with a NT_50_ titer of around 1:199 which is significantly higher than that of Ad5-JEV-E^WT^-immune mouse sera (NT_50_ titer is about 1:70, Figures 6A,B). Interestingly, sera from 6-week-old or 1-week-old mice immunized with JEV-E^WT^ mRNA-LNP or JEV-E^mut^ mRNA-LNP exhibit high level of JEV-neutralizing antibody response. The NT_50_ titers are 1:199 versus 1:933, 1:468 versus 1:588, respectively (Figures 6C–F). These results suggest that substitution of amino acid residues in the FL sequence of JEV does not affect the level of JEV-neutralizing antibody response induced by JEV-E^mut^.

### 3.6 JEV-E mRNA-LNP immunization protects mice against JEV challenge

**Figure 7:**
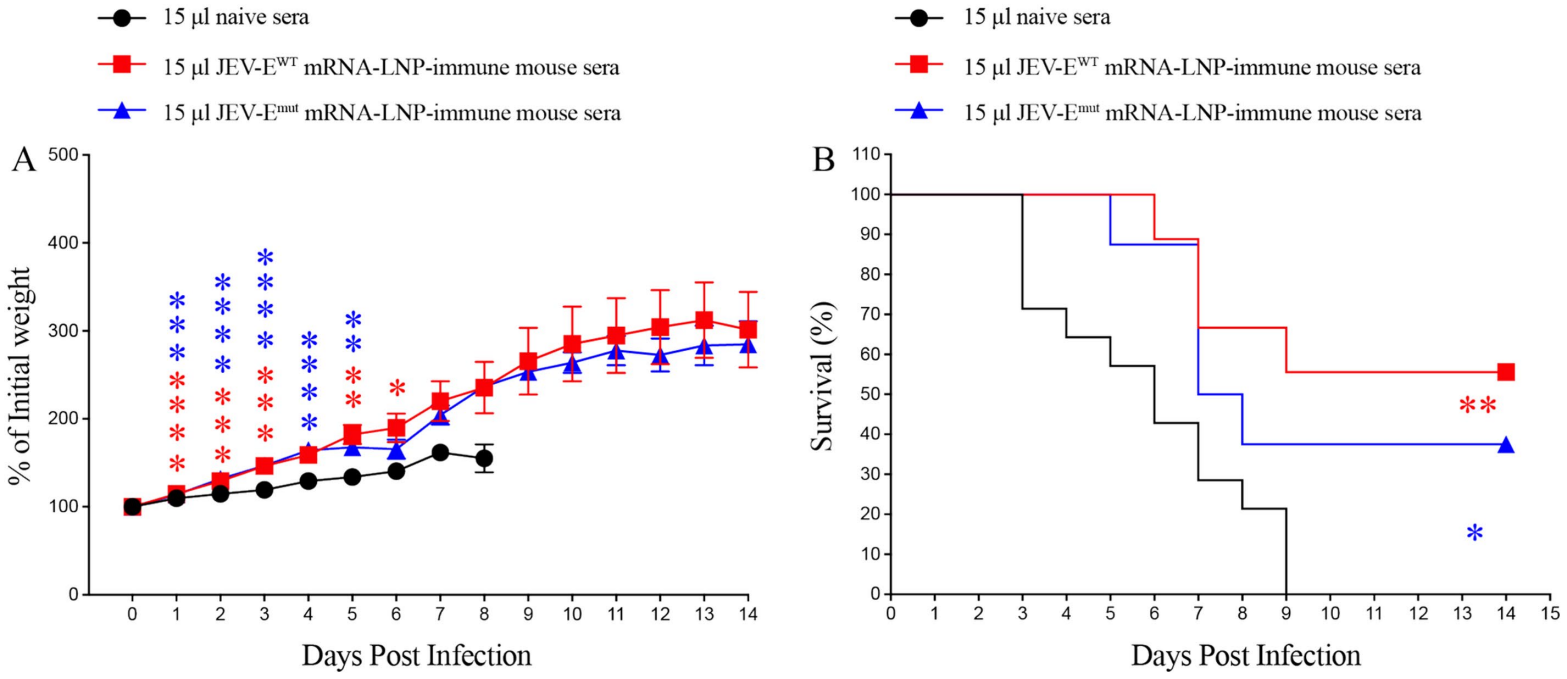
Transfer of JEV-E mRNA-LNP-immune mouse sera protects recipient mice against JEV attack. One-day-old naive C57BL/6 mice were injected s.c. with 15 μL of pooled mock-immune or JEV-E^WT^ mRNA-LNP-immune or JEV-E^mut^ mRNA-LNP-immune mouse sera. Two hours later, the mice were injected s.c. with 10 PFU of JEV. Mouse weights and survival were recorded daily for up to 14 days. Data are presented as the mean ± SEM. ^*^p < 0.05, ^**^p < 0.01, ^***^p < 0.001, and ^****^p < 0.0001 by two-tailed Mann–Whitney *U* test (A) or log-rank test (B).

To evaluate whether two types of JEV-E mRNA-LNP-immune mouse sera can protect recipient mice from JEV attack, we injected 15 μL of JEV-E^WT^ mRNA-LNP or JEV-E^mut^ mRNA-LNP-immune mouse sera into 1-day-old neonatal mice, and then attacked them with JEV, followed by mouse survival study. As shown in Figures 7A,B, the percent survival of recipient mice transferred with 15 μL of JEV-E^WT^ mRNA-LNP-immune mouse sera or JEV-E^mut^ mRNA-LNP-immune mouse sera was significantly higher than that of the control group (55.5% versus 0 and 37.5% versus 0, respectively).

**Figure 8:**
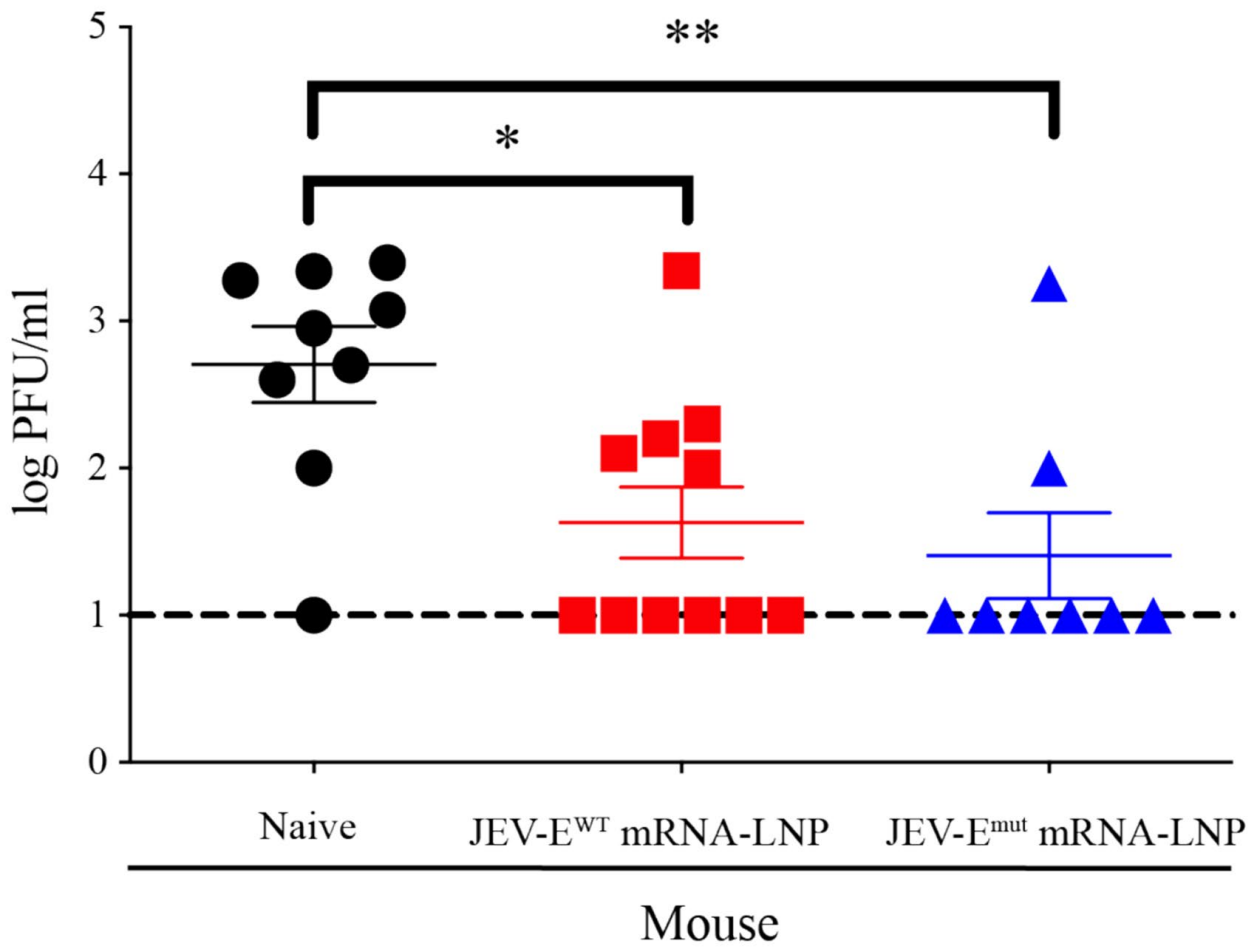
JEV-E mRNA-LNP immunization reduce the tissue viral loads of JEV-infected mice. One-week-old young mice were immunized with JEV-E mRNA-LNP as described in Materials and Methods. The immunized mice were challenged with 1 × 10^5^ PFU of JEV. Three days post infection, the mice were sacrificed and the mouse sera were collected for detecting viral load using PFA. Data are presented as the mean ± SEM. ^*^p < 0.05 and ^**^p < 0.01 by two-tailed Mann–Whitney *U* test.

Given the efficacy observed in passive serum transfer assays, whereas mouse-immune sera conferred protection against lethal JEV challenges, this next experiment aimed to ascertain whether our developed mRNA vaccine candidates could directly protect mice from JEV. To this end, one-week-old mice were immunized with 1 μg of JEV-E^WT^ mRNA or JEV-E^mut^ mRNA for twice and then challenged with JEV 2 weeks after the second immunization. Three days post JEV attack, the mouse sera were collected for viral load analysis. The data demonstrated that both mRNA-LNP immunization significantly shielded the mice from JEV infection. PFA results revealed that the levels of viral load in the sera of mice immunized with either JEV-E^WT^ mRNA-LNP or JEV-E^mut^ mRNA-LNP were substantially lower as compared to those in naïve mice (43 PFU/mL versus 501 PFU/mL, *p* = 0.0148; 26 PFU/mL versus 501 PFU/mL, *p* = 0.0093, respectively), confirming the protective efficacy of the JEV-E mRNA-LNP against JEV infection (Figure 8). These results indicate that mRNA constructed based on E protein mutant with optimized FL sequences showed no significant difference in protecting mice against ZIKV attacks compared to mRNA encoding wild-type E protein of JEV.

### 3.7 Transfer of JEV-E^mut^ mRNA-LNP-immune sera does not enhance ZIKV infection in recipient mice

**Figure 9:**
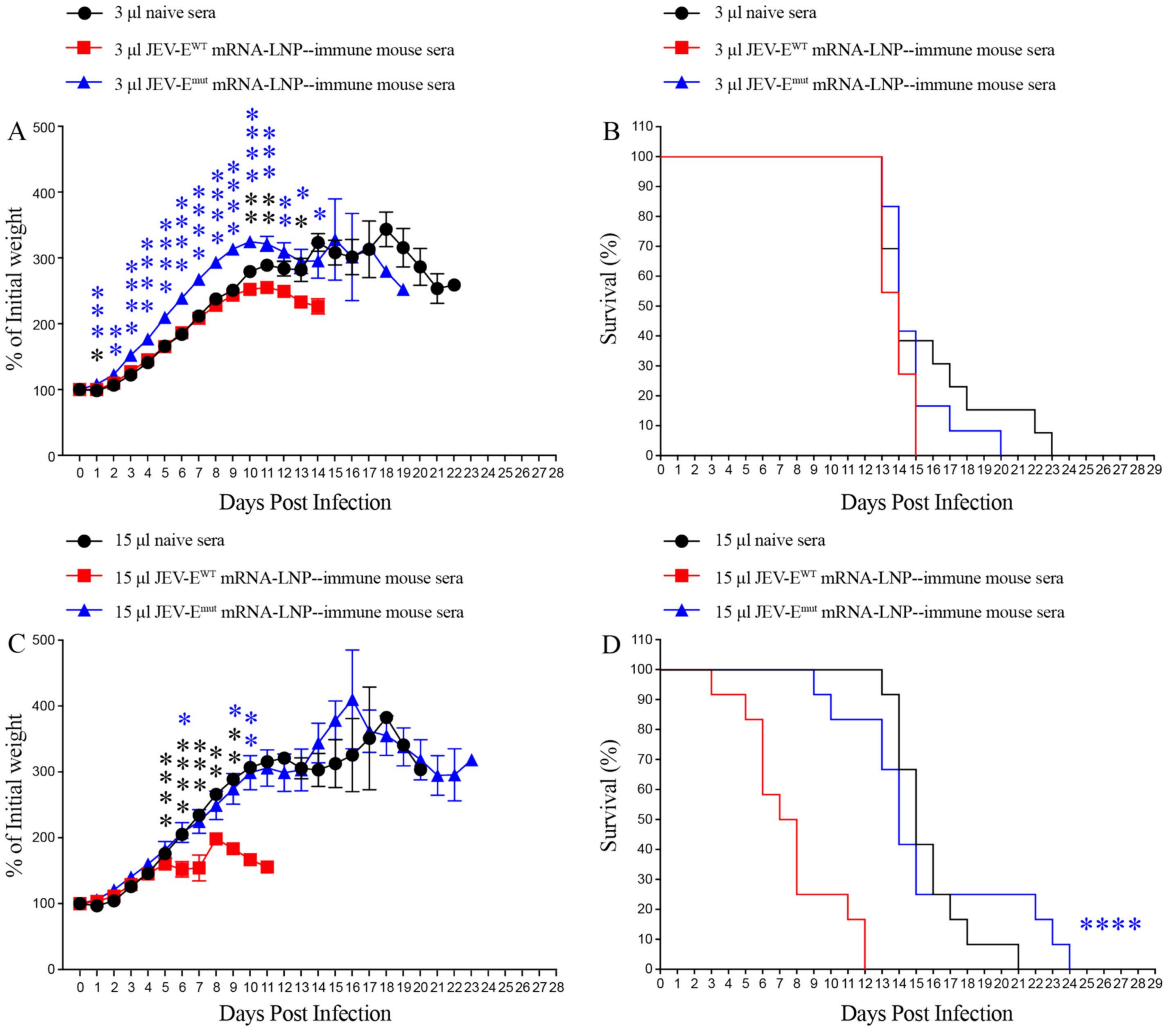
Transfer of JEV-E^mut^ mRNA-LNP-immune mouse sera do not accelerate the death of ZIKV-infected mice. One-day-old naive C57BL/6 mice were injected s.c. with 3 μL or 15 μL of pooled mock-immune or JEV-E^WT^ mRNA-LNP-immune or JEV-E^mut^ mRNA-LNP-immune mouse sera. Two hours later, the mice were injected s.c. with 1,000 PFU of ZIKV. Mouse weights and survival were recorded daily for up to 28 days. Data are presented as the mean ± SEM. ^*^p < 0.05, ^**^p < 0.01, ^***^p < 0.001, and ^****^p < 0.0001 by two-tailed Mann–Whitney *U* test (A,C) or log-rank test (B,D).

Considering a significant decrease in the level of ZIKV-cross-reactive antibody induced by JEV-E^mut^ mRNA-LNP, we next to explore whether the reduction of ZIKV-cross-reactive antibody response can potentially affect the occurrence of ADE of ZIKV infection *in vivo*. Specifically, we injected 3 or 15 μL of pooled JEV-E^WT^ mRNA-LNP- or JEV-E^mut^ mRNA-LNP-immune mouse sera into 1-day-old neonates and performed mouse survival study post ZIKV challenge. As a result, 1-day-old mice receiving 3 μL of pooled naive mouse sera all died within 22 days after ZIKV infection (Figures 9A,B). However, 1-day-old mice given with 3 μL of JEV-E^WT^ mRNA-LNP-immune mouse sera showed an accelerated death (all die within 14 days after ZIKV challenge) (Figures 9A,B). But, the 1-day-old mice transferred with 3 μL of JEV-E^mut^ mRNA-LNP-immune mouse sera all died at around 19 days after ZIKV infection and exhibited a significant reduction of weight loss compared with mice receiving naive mouse sera at multiple timepoints post ZIKV infection (Figures 9A,B).

Next, 1-day-old pups injected with 15 μL of naive mouse serum also died within 20 days after ZIKV infection. However, 1-day-old pups given with 15 μL of JEV-E^WT^ mRNA-LNP-immune mouse serum exhibited a significant acceleration of death (all died within 11 days after ZIKV infection). However, 1-day-old pups receiving 15 μL of JEV-E^mut^ mRNA-LNP-immune mouse serum died around 23 days after ZIKV infection. Compared to JEV-E^mut^ mRNA-LNP-immune mouse serum, JEV-E^WT^ mRNA-LNP-immune mouse serum significantly accelerated the death of 1-day-old mice (Figures 9C,D). These results indicate that the Abs induced by mRNA encoding wild-type E protein of JEV can exacerbate ZIKV infection in mice at the specific doses, leading to earlier death of mouse, representing speculated ADE of ZIKV infection. The Abs induced by mRNA encoding the E protein mutant of JEV did not have an obvious enhancing effect on ZIKV infection, suggesting optimized JEV mRNA vaccine is safer than unmodified mRNA vaccine.

### 3.8 Maternally acquired ZIKV-reactive Ab did not increase the mortality of pups born to mRNA-LNP-immune mice

**Figure 10:**
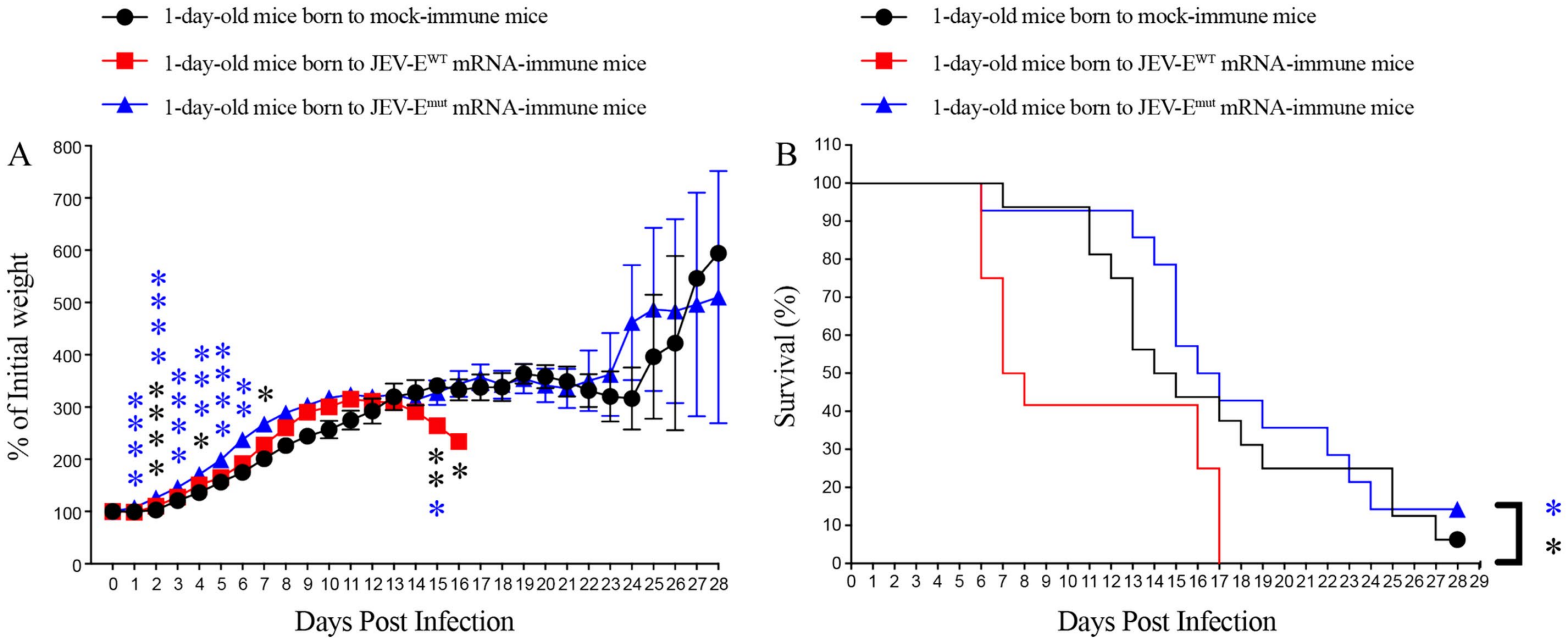
Maternally acquired ZIKV-reactive antibody did not increase the mortality of pups born to mRNA-LNP-immune mice. Six-week-old female C57BL/6 mice were immunized with JEV-E^WT^ mRNA-LNP or JEV-E^mut^ mRNA-LNP as described in materials and methods. Four weeks later, mock-immune or JEV-E mRNA-LNP-immune females were mated with 10-week-old naive male mice. One-day-old pups born to JEV-E mRNA-LNP-immune females were injected s.c. with 1,000 PFU of ZIKV. Mouse weights and survival were recorded daily for up to 28 days. Data are presented as the mean ± SEM. ^*^p < 0.05, ^**^p < 0.01, ^***^p < 0.001, and ^****^p < 0.0001 by two-tailed Mann–Whitney *U* test (A) or log-rank test (B).

Although our passive transfer experiment results showed that Abs against the JEV-E^WT^ protein can accelerate the death of ZIKV-infected mice at certain doses, but, do anti JEV-E^WT^ Abs have ADE against ZIKV infection in the real world? If so, whether the Abs induced by optimized mRNA vaccine will not mediate ADE of ZIKV infection? Considering that IgG in the mother’s body can across the placenta and enter the body of fetus or newborn, as well as that many women who have been infected with JEV or vaccinated with JEV vaccine will have children who may subsequently be infected with ZIKV, we then evaluate the impact of anti-JEV Abs obtained by the pups from the female mice vaccinated with either JEV-E^WT^ mRNA-LNP or JEV-E^mut^ mRNA-LNP on subsequent ZIKV infection. The results showed that the 1-day-old mice born to JEV-E^mut^ mRNA-LNP-immunized female mice loss less weight compared to 1-day-old mice born to naive females post ZIKV challenge (Figure 10A). Approximately 6% of 1-day-old mice born to naive female mice survived ZIKV infection, while all 1-day-old mice born to JEV-E^WT^ mRNA-LNP-immunized female mice died within 17 days post ZIKV challenge. In comparison, around 14% of 1-day-old mice born to JEV-E^mut^ mRNA-LNP-immunized female mice survived ZIKV infection (Figure 10). These results suggest that maternally acquired Abs from JEV-E^WT^ mRNA-LNP-immunized female mice can accelerate the death of 1-day-old mice during ZIKV infection, while the maternally acquired Abs against JEV-E^mut^ protein do not exacerbate the death of 1-day-old mice infected with ZIKV, but rather slightly improve the percent survival of mice, suggesting that Abs induced by JEV-E^mut^ mRNA immunization may not make the host develop severe diseases after ZIKV infection.

## 4 Discussion

Fusion loop sequence (98-111 aa of E protein) is a highly conserved sequence among many flaviviruses (Supplementary Table S3). The FL sequence (DRGWGNGCGLFGKG) of DENV is same to that of ZIKV while there is one amino acid residue variation (L108F) in the FL sequence of JEV (DRGWGNGCGFFGKG). FL has strong immunogenicity and responsible for the induction of flavivirus-cross-reactive Abs (de Alwis et al., 2014; Gunawardana and Shaw, 2018; Vogt et al., 2011). In the present study, we replaced the amino acid W at position 101 and the amino acid F at position 108 in the FL sequence of JEV with amino acid A and obtain an E^mut^ protein sequence. We then constructed the recombinant adenovirus and mRNA-LNP encoding JEV-E^WT^ and JEV-E^mut^. Our *in vitro* and *in vivo* experimental results showed that the levels of JEV-neutralizing Abs induced by either Ad5-JEV-E^mut^ or JEV-E^mut^ mRNA-LNP did not decrease as compared with vaccine candidates encoding JEV-E^WT^, while the levels of Abs reactive to ZIKV antigen and its ADE effect on ZIKV infection were significantly reduced. Considering that our and other research groups’ previous results indicate that JEV infection- or vaccine inoculation-induced Abs have an ADE effect on ZIKV infection (Chen et al., 2020; He et al., 2020; Zhang et al., 2020), thus, optimized JEV vaccines may have broad application value in the future.

In the past several decade years, a growing number of studies have highlighted that ADE phenomenon is commonly present between different serotypes of the same flavivirus [such as dengue virus (DENV)], as well as between various flavivirus species (Bardina et al., 2017; Chen et al., 2020; He et al., 2020; Zhang et al., 2020; Katzelnick et al., 2017; Katzelnick et al., 2020). Considering that most flavivirus cross-reactive Abs are produced against the highly conserved FL domain in the viral E protein, researchers have made some attempts to optimize the FL sequence, and have achieved some expected results (Berneck et al., 2020; Zhang et al., 2006; Rockstroh et al., 2015). An earlier study showed that a single substitution (L107F) within the FL of West Nile virus abolished binding ability of a non-neutralizing Mab (3D9) which is broadly cross-reactive with other flaviviruses (Zhang et al., 2006). Later, Rockstroh et al. (2015), generated recombinant E proteins which contain four mutations (T76R, Q77E, W101R, L107R) in the FL domain of four DENV serotypes. The specific mixtures of these antigen mutants exhibit reduced cross-reactivity against heterologous flaviviruses. Berneck et al. (2020), expressed an E protein mutant carrying four mutations (T76A, Q77G, W101R and L107R) in and near the FL of ZIKV and observed that this antigen mutant elicits Abs with equal neutralizing capacity as the wildtype E protein. But, this mutant antigen induces much less serological cross-reactivity and does not cause ADE of DENV infection.

In addition, although two studies identified that DENV virus-like particles carrying W101A and F108A substitutions were not recognized by cross-reactive antibodies, but the researchers did not evaluate whether the capacity of this DENV VLP mutant to induce ADE-mediating antibodies will be attenuated (Lai et al., 2013; Tsai et al., 2013). Moreover, whether these substitutions (W101A and F108A) affect the production of neutralizing antibodies also needs to be considered. For instance, researchers introduced four amino acid mutations (T76R, Q77E, W101R, and L107R) into the E protein of ZIKV and prepared mRNA-LNP encoding prM-E. The ADE effect of the vaccine-induced antibodies on DENV infection was significantly attenuated, but the level of ZIKV-neutralizing antibodies induced by the vaccine was also weakened (Richner et al., 2017). In the present study, we designed an E protein mutant by introducing W101A and F108A substitutions in the FL sequence of the JEV viral E protein and constructed recombinant adenovirus and mRNA vaccine because these two types of vaccines can induce strong humoral and cellular immunity. In the preparation of mRNA vaccines, we prioritize choosing codons rich in adenine (A) or guanine (G) to replace codons containing cytidine (C) and uridine (U), as this strategy can increase the stability of RNA. As expected, these substitutions we introduced in the FL sequence of JEV did not affect the production of JEV-neutralizing antibodies, but significantly reduced the production of ZIKV-cross-reactive antibodies and attenuated the ADE of ZIKV infection. These results indicate that optimizing the amino acid sequence of the FL sequence of the flavivirus has the potential to reduce the potential ADE of the vaccine against heterologous flavivirus infections. Previous studies showed that CD8^+^ T cell response induced by JEV infection or vaccine inoculation plays a protective activity against JEV and ZIKV infection (Chen et al., 2020; Zhang et al., 2020). In this study, we found that JEV-E mRNA-LNP immunization could induce IFNγ-secreting T cell response reactive with both JEV and ZIKV peptides. Thus, vaccines constructed using this strategy should have safe profile and have broad application scenarios in many countries/territories, especially in areas where multiple flaviviruses are co-circulating.

Recombinant adenovirus is better than recombinant protein in immune response especially cellular immune response, a property that underscores the feasibility of vaccines based on recombinant adenovirus vectors, as evidenced by extensive human trials. Owing to their non-replicating nature within the host organism, recombinant adenovirus vaccines minimize safety concerns, thereby offering a promising avenue for both prophylactic and therapeutic immunization strategies. Recombinant adenovirus vaccine is currently extensively studied in the prevention of many infectious diseases, including COVID-19, chikungunya fever, and hepatitis C (Agrawal et al., 2019; Lyke et al., 2022; Cao et al., 2022). An mRNA vaccine is a vaccine that prevents or treats a disease by introducing into the body a mRNA that expresses a specific antigen, thereby inducing an immune response against the pathogen or antigenic protein. The unique advantages of mRNA technology are as follows: firstly, due to the standardized process of mRNA vaccine production, rapid development of a new vaccine against emerging pathogen is feasible by simply substituting the mRNA sequence specific to the specific pathogen. This makes the design and production of mRNA vaccines more convenient and cost-effective compared to traditional vaccines (Schlake et al., 2012; Pardi et al., 2018; Linares-Fernandez et al., 2020). Secondly, mRNA sequence inherently possesses adjuvant properties because it can activate innate immune responses to produce cytokines, thereby assisting adaptive immune response. In contrast, traditional protein-based vaccines require additional adjuvants to achieve similar effects (Iavarone et al., 2017). Thirdly, mRNA vaccines, devoid of live viruses, eliminate the risk of infection in vaccine recipients. Moreover, the chemical structure of mRNA, being distinct from DNA, minimizes the likelihood of integration into the host’s DNA genome (Sahin et al., 2014). Given that mRNA is inherently susceptible to degradation by nucleases, and further compounded by its propensity for self-hydrolysis, naked mRNA exhibits instability. Encapsulating mRNA within LNPs serves to shield it from nuclease degradation, concurrently facilitating the delivery of mRNA.

Developing engineered vaccines based on mutations in the FL sequence of the E protein of the JEV is of paramount importance for several reasons. Firstly, there is a considerable geographic overlap between the endemic regions of JEV and ZIKV. In these epidemic regions (particularly Southeast Asian and East Asian countries/territories), the majority of the population has already been infected with JEV or vaccinated with JEV vaccine, and the Abs induced by JEV may have potential ADE effects on subsequent ZIKV infections (Chen et al., 2020). The antibodies generated from JEV vaccination persist in the body longer than the duration of cellular immunity (Wang et al., 2020), which may increase the risk of ADE in individuals infected with heterologous flavivirus. Therefore, it is necessary to optimize the present JEV vaccine. Secondly, individuals with compromised immune systems, such as HIV patients, exhibit weaker cellular immunity, rendering them more susceptible to severe ZIKV infections.

## 5 Conclusion

In summary, our results indicate that indicated amino acid substitutions within the FL region of the JEV E protein exert a negligible impact on the production of JEV-neutralizing Abs, but, significantly reduce the levels of cross-reactive Abs against ZIKV, thereby serving as a strategic approach in the development of safer JEV vaccines.

## Data Availability

The original contributions presented in the study are included in the article/Supplementary material, further inquiries can be directed to the corresponding author.
