# Supplementary material for: An engineered Japanese encephalitis virus mRNA-lipid nanoparticle immunization induces protective immunity in mice
Source: Front Microbiol. 2024 Nov 11;15:1472824. doi: 10.3389/fmicb.2024.1472824 (PMC11586386; doi:10.3389/fmicb.2024.1472824)
Supplement: Supplementary file 1 [file Data_Sheet_1.docx]

**An engineered Japanese encephalitis virus mRNA-lipid nanoparticle immunization induces protective immunity in mice**

Jiayang Zhu^1, a^, Caiying He^1, a^, Yusha Liu^1, a^, Min Chen^1^, Jiayi Zhang^1, 2, 3^, Dong Chen^1, 2, 3^, Hongxia Ni^4^, Jinsheng Wen ^1^*

^1^ School of Basic Medical Sciences, Health Science Center, Ningbo University, Ningbo, China
^2^ Wenzhou Central Blood Station, Wenzhou, China
^3^ Key Laboratory of Laboratory Medicine, Ministry of Education, Zhejiang Provincial Key Laboratory of Medical Genetics, College of Laboratory Medicine and Life sciences, Wenzhou Medical University, Wenzhou, China

^4^ Ningbo Municipal Center for Disease Control and Prevention, Ningbo, China

^a^These authors contributed equally to this work.

*CORRESPONDENCE. Jinsheng Wen, wenjinsheng@nbu.edu.cn

**Supplementary materials**

**
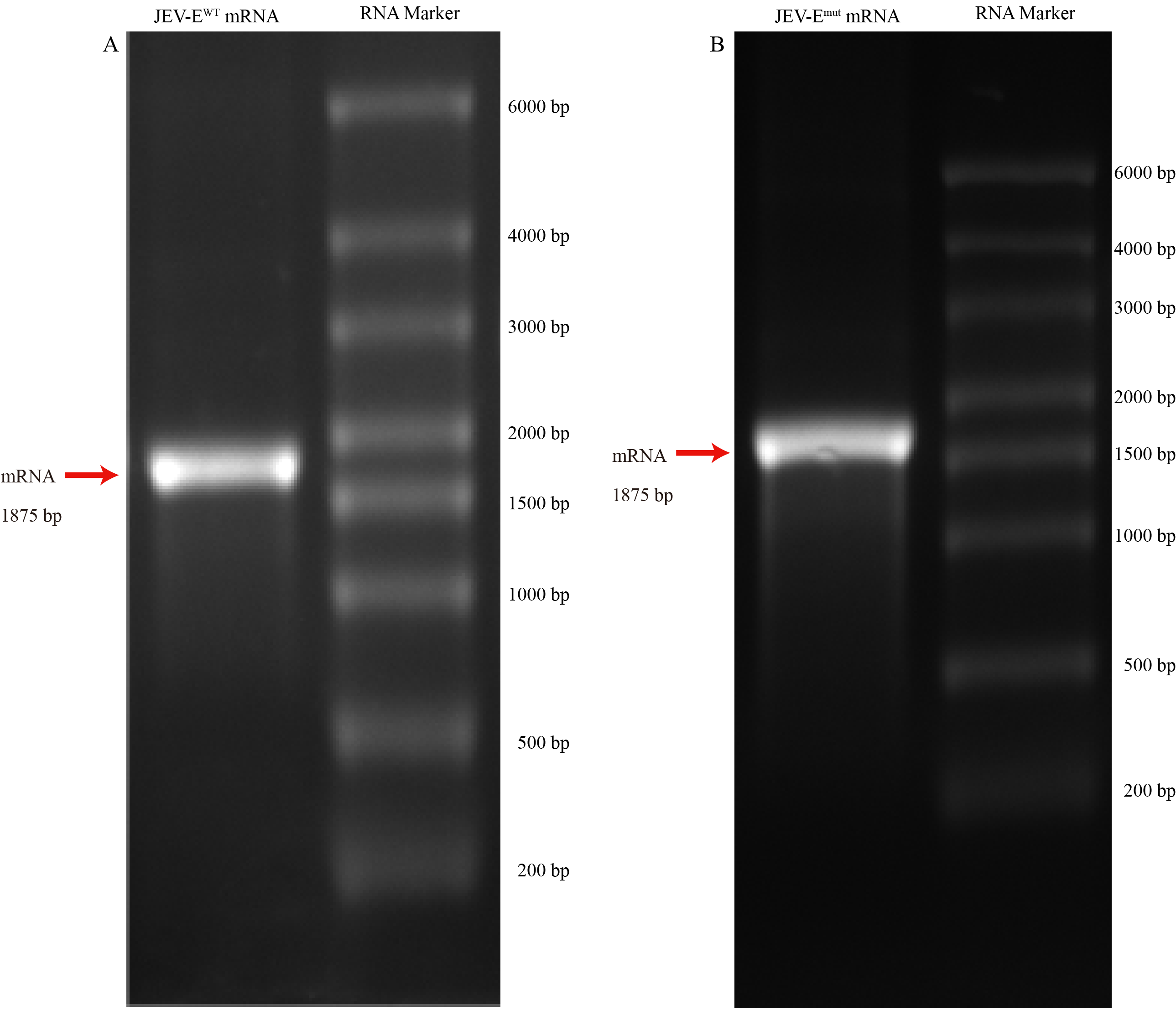
**

**FIGURE S1.** Agarose gel electrophoresis of mRNA prepared *in vitro*

Using linearized recombinant plasmid as template, RNA was prepared by *in vitro* transcription. mRNA was generated by capping the 5' terminus of RNA and subjected to agarose gel electrophoresis.


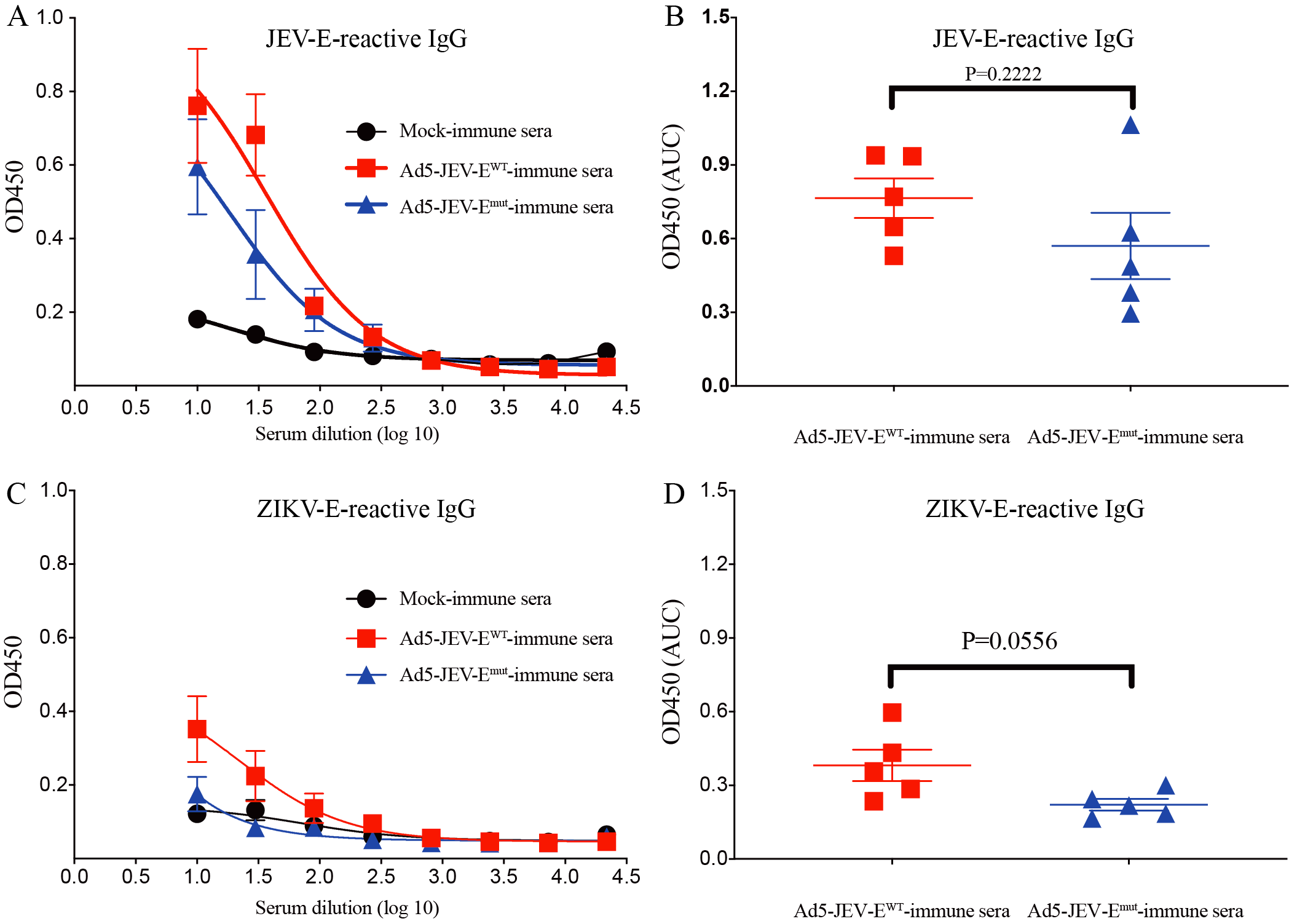


**FIGURE S2**. Ad5-JEV-E^mut^ immunization induces low level of ZIKV-E-cross-reactive IgG response

(A-D) Mouse sera were collected from mock-immune (n=5), Ad5-JEV-E^WT^-immune (n=5), and Ad5-JEV-E^mut^-immune mice (n=5). The levels of JEV-E_406_- and ZIKV-E_410_-reactive IgG were measured using ELISA. Data are presented as the mean ± SEM.

| **TABLE S1.** JEV-derived H-2^b^-restricted epitopes and ZIKV variants | | |
| --- | --- | --- |
| Peptides^a^ | Sequences | Homology^b^ |
| JEV-E_4-12_ | LGMGNRDFI | 56% |
| ZIKV-E_4-12_ | IGVSNRDFV |  |
| JEV-E_57-65_ | RSYCYHASV | 78% |
| ZIKV-E_57-65_ | RSYCYEASI |  |
| JEV-E_416-424_ | RLAALGDTA | 78% |
| ZIKV-E_420-428_ | RMAVLGDTA |  |
| ^a^ Peptide position is determined according to the amino acid sequence JEV strain and ZIKV strain. | | |
| ^b^ The percentage of shared amino acid between ZIKV epitope and JEV variant. | | |

**Table S2. The sequences of the DNA template for producing mRNA**

Poly (A)

3' UTR

JEV-E

tPA signal peptide

Kozak sequence

5' UTR

T7 promoter

5'- -3'

5'–**taatacgactcactatagggcgaattgacatttgcttctgacacaactgtgttcactagcaacctcaaacagacaccgccaccatggacgctatgaagaggggcctgtgctgtgtgctgctgctgtgcggagctgtgtttgtgtcc [JEV-E^WT^ or JEV-E^mut^ sequence] gctcgctttcttgctgtccaatttctattaaaggttcctttgttccctaagtccaactactaaactgggggatattatgaagggccttgagcatctggattctgcctaataaaaaacatttattttcattgcaaaaaaaaaaaaaaaaaaaaaaaaaaaaaaaaaaaaaaaaaaaaaaaaaaaaaaaaaaaaaaaaaaaaaaaaaaaaaaaaaaaaaaaaaaaaaaaaaaaaaaaaaaaaaaaaaaaa**–3'

**(1) JEV-E^WT^
5'–TTCAACTGCCTGGGAATGGGAAACCGAGACTTCATAGAGGGAGCAAGCGGAGCAACATGGGTGGACCTGGTGCTGGAGGGAGACAGCTGCCTGACAATAATGGCAAACGACAAGCCAACACTGGACGTGCGAATGATAAACATAGAGGCAAGCCAGCTGGCAGAGGTGCGAAGCTACTGCTACCACGCAAGCGTGACAGACATAAGCACAGTGGCACGATGCCCAACAACAGGAGAGGCACACAACGAGAAGCGAGCAGACAGCAGCTACGTGTGCAAGCAGGGCTTCACAGACCGAGGATGGGGAAACGGATGCGGATTCTTCGGAAAGGGAAGCATAGACACATGCGCAAAGTTCAGCTGCACAAGCAAGGCAATAGGCCGAACAATACAGCCAGAGAACATAAAGTACAAGGTGGGAATATTCGTGCACGGAACAACAACAAGCGAGAACCACGGAAACTACAGCGCACAGGTGGGAGCAAGCCAGGCAGCAAAGTTCACAGTGACACCAAACGCACCAAGCGTGGCACTGAAGCTGGGAGACTACGGAGAGGTGACACTGGACTGCGAGCCACGAAGCGGACTGAACACAGAGGCATTCTACGTGATGACAGTGGGAAGCAAGAGCTTCCTGGTGCACCGAGAGTGGTTCCACGACCTGGCACTGCCATGGACAAGCCCAAGCAGCACAGCATGGCGAAACCGAGAGCTGCTGATGGAGTTCGAGGGAGCACACGCAACAAAGCAGAGCGTGGTGGCACTGGGAAGCCAGGAGGGAGGACTGCACCACGCACTGGCAGGAGCAATAGTGGTGGAGTACAGCAGCAGCGTGATGCTGACAAGCGGACACCTGAAGTGCCGACTGAAGATGGACAAGCTGGCACTGAAGGGAACAACATACGGAATGTGCACAGAGAAGTTCAGCTTCGCAAAGAACCCAGTGGACACAGGACACGGAACAGTGGTGATAGAGCTGAGCTACAGCGGAAGCGACGGACCATGCAAGATACCAATAGTGAGCGTGGCAAGCCTGAACGACATGACACCAGTGGGACGACTGGTGACAGTGAACCCATTCGTGGCAACAAGCAGCGCAAACAGCAAGGTGCTGGTGGAGATGGAGCCACCATTCGGAGACAGCTACATAGTGGTGGGACGAGGAGACAAGCAGATAAACCACCACTGGCACAAGGCAGGAAGCACACTGGGAAAGGCATTCAGCACAACACTGAAGGGAGCACAGCGACTGGCAGCACTGGGAGACACAGCATGGGACTTCGGAAGCATAGGAGGAGTGTTCAACAGCATAGGACGAGCCGTGCACCAGGTGTTCGGAGACGCATTCCGAACACTGTTCGGAGGAATGAGCTGGATAACACAGGGACTGATGGGAGCACTGCTGCTGTGGATGGGAGTGAACGCACGAGACCGAAGCATAGCACTGGCATTCCTGGCAACAGGAGGAGTGCTGGTGTTCCTGGCAACAAACGTGCACGCATga–3'**

**(2) JEV-E^mut^**

**5'–**

**TTCAACTGCCTGGGAATGGGAAACCGAGACTTCATAGAGGGAGCAAGCGGAGCAACATGGGTGGACCTGGTGCTGGAGGGAGACAGCTGCCTGACAATAATGGCAAACGACAAGCCAACACTGGACGTGCGAATGATAAACATAGAGGCAAGCCAGCTGGCAGAGGTGCGAAGCTACTGCTACCACGCAAGCGTGACAGACATAAGCACAGTGGCACGATGCCCAACAACAGGAGAGGCACACAACGAGAAGCGAGCAGACAGCAGCTACGTGTGCAAGCAGGGCTTCACAGACCGAGGAGCAGGAAACGGATGCGGATTCGCAGGAAAGGGAAGCATAGACACATGCGCAAAGTTCAGCTGCACAAGCAAGGCAATAGGCCGAACAATACAGCCAGAGAACATAAAGTACAAGGTGGGAATATTCGTGCACGGAACAACAACAAGCGAGAACCACGGAAACTACAGCGCACAGGTGGGAGCAAGCCAGGCAGCAAAGTTCACAGTGACACCAAACGCACCAAGCGTGGCACTGAAGCTGGGAGACTACGGAGAGGTGACACTGGACTGCGAGCCACGAAGCGGACTGAACACAGAGGCATTCTACGTGATGACAGTGGGAAGCAAGAGCTTCCTGGTGCACCGAGAGTGGTTCCACGACCTGGCACTGCCATGGACAAGCCCAAGCAGCACAGCATGGCGAAACCGAGAGCTGCTGATGGAGTTCGAGGGAGCACACGCAACAAAGCAGAGCGTGGTGGCACTGGGAAGCCAGGAGGGAGGACTGCACCACGCACTGGCAGGAGCAATAGTGGTGGAGTACAGCAGCAGCGTGATGCTGACAAGCGGACACCTGAAGTGCCGACTGAAGATGGACAAGCTGGCACTGAAGGGAACAACATACGGAATGTGCACAGAGAAGTTCAGCTTCGCAAAGAACCCAGTGGACACAGGACACGGAACAGTGGTGATAGAGCTGAGCTACAGCGGAAGCGACGGACCATGCAAGATACCAATAGTGAGCGTGGCAAGCCTGAACGACATGACACCAGTGGGACGACTGGTGACAGTGAACCCATTCGTGGCAACAAGCAGCGCAAACAGCAAGGTGCTGGTGGAGATGGAGCCACCATTCGGAGACAGCTACATAGTGGTGGGACGAGGAGACAAGCAGATAAACCACCACTGGCACAAGGCAGGAAGCACACTGGGAAAGGCATTCAGCACAACACTGAAGGGAGCACAGCGACTGGCAGCACTGGGAGACACAGCATGGGACTTCGGAAGCATAGGAGGAGTGTTCAACAGCATAGGACGAGCCGTGCACCAGGTGTTCGGAGACGCATTCCGAACACTGTTCGGAGGAATGAGCTGGATAACACAGGGACTGATGGGAGCACTGCTGCTGTGGATGGGAGTGAACGCACGAGACCGAAGCATAGCACTGGCATTCCTGGCAACAGGAGGAGTGCTGGTGTTCCTGGCAACAAACGTGCACGCATga–3'**

| TABLE S3. Sequence alignment of fusion loops of multiple flaviviruses | |
| --- | --- |
| Flaviviruses | Fusion loops^a^ |
| Dengue virus serotype 1 | DRGWGNGCGLFGKG |
| Dengue virus serotype 2 | DRGWGNGCGLFGKG |
| Dengue virus serotype 3 | DRGWGNGCGLFGKG |
| Dengue virus serotype 4 | DRGWGNGCGLFGKG |
| Japanese encephalitis virus | DRGWGNGCGFFGKG |
| Zika virus | DRGWGNGCGLFGKG |
| Bagaza virus | DRGWGNGCGLFGKG |
| Gadgets gully virus | DRGWGNHCGLFGKG |
| Kokobera virus | DRGWGNGCGLFGKG |
| Kyasanur forest disease virus | DRGWGNHCGLFGKG |
| Langat virus | DRGWGNHCGLFGKG |
| Louping ill virus | DRGWGNHCGLFGKG |
| Murray valley encephalitis virus | DRGWGNGCGLFGKG |
| Powassan virus | DRGWGNHCGFFGKG |
| Saint Louis encephalitis virus | DRGWGNGCGLFGKG |
| Saumarez reef virus | DRGWGNHCGLFGKG |
| Tembusu virus | DRGWGNGCGLFGKG |
| Tick-borne encephalitis virus | DRGWGNHCGLFGKG |
| Usutu virus | DRGWGNGCGLFGKG |
| Wesselsbron virus | DRGWGNGCGLFGKG |
| West Nile virus | DRGWGNGCGLFGKG |
| Yellow fever virus | DRGWGNGCGLFGKG |
| Kunjin virus | DRGWGNGCGLFGKG |
| Alfuy virus | DRGWGNGCGLFGKG |
| New Mapoon virus | DRGWGNGCGLFGKG |
| Rocio virus | DRGWGNGCGLFGKG |
| ^a^ The amino acid variants were highlighted in red. | |
